# Mathematical Modeling of Physical Reality: From Numbers to Fractals, Quantum Mechanics and the Standard Model

**DOI:** 10.3390/e26110991

**Published:** 2024-11-18

**Authors:** Marian Kupczynski

**Affiliations:** Département de l’Informatique et d’Ingénierie, Université du Québec en Outaouais (UQO), Case Postale 1250, Succursale Hull, Gatineau, QC J8X 3X7, Canada; marian.kupczynski@uqo.ca

**Keywords:** physical reality, perceptions and neuroscience, Bild conception, causality, mathematical modeling, classical mechanics, chaos theory, fractals, quantum mechanics, Standard Model

## Abstract

In physics, we construct idealized mathematical models in order to explain various phenomena which we observe or create in our laboratories. In this article, I recall how sophisticated mathematical models evolved from the concept of a number created thousands of years ago, and I discuss some challenges and open questions in quantum foundations and in the Standard Model. We liberated nuclear energy, landed on the Moon and built ‘quantum computers’. Encouraged by these successes, many believe that when we reconcile general relativity with quantum theory we will have the correct theory of everything. Perhaps we should be much humbler. Our perceptions of reality are biased by our senses and by our brain, bending them to meet our priors and expectations. Our abstract mathematical models describe only in an approximate way different layers of physical reality. To describe the motion of a meteorite, we can use a concept of a material point, but the point-like approximation breaks completely when the meteorite hits the Earth. Similarly, thermodynamic, chemical, molecular, atomic, nuclear and elementary particle layers of physical reality are described using specific abstract mathematical models and approximations. In my opinion, the theory of everything does not exist.

## 1. Introduction

Physical reality is a complex concept which has been discussed by philosophers and physicists for centuries. For us, it represents everything which exists independently of human perceptions or beliefs. Our perceptions are biased by our senses and by our brain to meet our priors and expectations. Nevertheless, we discovered different layers of physical reality and succeeded in describing them using specific abstract mathematical models and approximations. This is why, Gauss said: “*mathematics is the queen of science, and arithmetic the queen of mathematics*”. However, physical reality is much more than abstract mathematical models we create to describe it and, in general, these models do not allow for far-reaching metaphysical speculations.

In this article, we review how different physical and mathematical concepts and models evolved through the centuries, starting from a primitive notion of causality and how it has been used until today. We discuss also some challenges and open questions in the Standard Model and in the foundations of quantum mechanics.

As soon as we are born, we learn that our actions and our parents’ actions have consequences. If we cry, we are fed, covered, cuddled or cleaned. If we open our eyes, we see the external world. If we notice a toy, we have to move our hand to grasp it or have to crawl or walk before retrieving it. This is how we acquire a basic notion of causality by which one event contributes to the occurrence of another event. From early childhood, we are asking a question, ‘‘Why…’’, and we are receiving answers, ‘‘Because…’’, but to any answer ‘‘Because…’’ there is immediately another question ‘‘Why…?’’ and so on.

Causality is probably the most fundamental notion which any living organism had to understand in order to survive. Any action has a consequence and what is happening around them may have an immediate or subsequent impact on the organism’s well-being and fate.

We agree with Robb [1] and Whitehead [2] that the notion of causality is prior to notions of time and space because it is necessary for the interpretation of observations and empirical experiments.

In any place on Earth, there are specific diurnal, monthly and yearly patterns, the Sun and the Moon are moving, seasons are changing, animals mate, give birth, migrate and die. Moreover, man has always been searching for the answer to the following questions: How did the universe come about? What happens after death? Is there a plan for the solar system? What causes light? [3].

There existed curious individuals, later called astronomers, philosophers, mathematicians and scientists, who believed that observed periodic natural patterns reflect an intelligent causal design of the universe. Therefore, they observed and recorded how the Sun, Moon and planets were moving and searched for an explanation. Such explanations became possible due to the study of the properties of numbers by Pythagoreans in 6th century BC, followed by the creation of arithmetic, logic and abstract geometry by the Greeks.

These efforts led to the fundamental findings of Euclidean geometry, still taught in our schools, to Aristotelian principles of logical reasoning, still used in courts, and to Ptolemy’s quite precise geocentric planetary model, which survived 15 centuries before being replaced by the Copernican and Kepler’s heliocentric model.

Copernicus and Kepler were searching for a systematic harmonious mathematical model which should please the God creator. Kepler, who was a mystic and an astrologer, after discovering his three laws governing the motion of planets in their elliptical orbits concluded in Harmony of the World (1619), ‘*The wisdom of the Lord is infinite; so also are his Glory and His power’*. He believed that the different angular velocities of planets are arranged to play music for God. In fact, this belief helped him to discover his laws [3].

Galileo, Newton, Leibniz, Euler, Gauss, Descartes, Spinoza, Kant, Darwin, Einstein rejected many religious dogmas but strongly believed in the intelligent divine design of the universe. Darwin’s religious views evolved from Christian orthodoxy to an agnostic stance.

For Einstein, the problem of God transcended humans’ limited understanding; nevertheless, he admitted, “I believe in Spinoza’s God, who reveals himself in the orderly harmony of what exists, not in a God who concerns himself with the fates and actions of human beings”. He believed that “God does not play dice” and that quantum theory cannot be considered as a complete theory of physical reality.

Our perceptions of reality are biased, this is why several philosophers and scientists pointed out that our models describe physical reality as we perceive it and not as it is.

Emmanuel Kant strongly insisted that our knowledge is limited to the realm of empirical phenomena and that the nature of things as they are in themselves (i.e., beyond our perceptual experience) remains unknowable. Nevertheless, the human mind supplies the concepts and axioms building up reliable knowledge with the sensations it receives [3].

In 1878, von Helmholtz posed the following philosophical questions [4,5]: “What is true in our intuition and thought? In what sense do our representations correspond to actuality”? He criticized the objective conception of physical theory. In his Bild conception, physical theory is only an intellectual construct of our brain, “In as much as the quality of our sensation gives us a report of what is peculiar to the external influence by which it is excited, it may count as a symbol of it, but not as an image…” [4].

The Bild conception was further developed and promoted by Hertz [6,7], Boltzmann [8], Schrodinger [9,10] and was reviewed by Agostino [11] and Khrennikov [12].

Laplace believed that with classical mechanics and probability theory, man is capable of explaining the causes and laws governing the universe. Many contemporary physicists also believe that if we succeed in reconciling the general theory of relativity with the quantum field theory we will obtain the final theory of everything.

It is true that the successes of modern science and technology are impressive, but we should be much humbler. The theory of everything does not exist.

Our article is organized as follows. In Section 2 we discuss the numerical abilities of living species and recall some successes of Babylonian and Egyptian practical mathematics. In Section 3, we explain how Greeks laid the fundaments of modern science by creating an abstract concept of numbers, Euclidean geometry and Ptolemy’s planetary model. In Section 4, we resume with Copernican revolution and Newtonian classical mechanics. In Section 5, we discuss the Three-Body-Problem and chaos theory. Section 6 is about fractal geometry in nature. In Section 7 and Section 8, we retrace the evolution of atomistic ideas from Democritus to quantum mechanics. In Section 9, we discuss open questions in quantum field theory, in elementary particle physics and in the Standard Model. Section 10 is about the Bild conception of physical reality and how it is supported by modern neuro-science. Section 11 contains several conclusions.

## 2. A Short History of Numbers: Babylonian and Egyptian Mathematics

Homo sapiens evolved in Africa approximately 300,000 to 200,000 years ago from their early predecessors. Their important capacity for language developed around 50,000 years ago or earlier. During the 4th millennium BC, Sumerians developed cuneiform writing on clay tablets to represent spoken language and the Egyptians started to use hieroglyphs. Chinese writing developed around 1400 BC. The invention of writing marked an important turning point in human history because it allowed the transfer of culture, acquired skills and knowledge to the next generations.

Different animal species have different sensorial organs to explore their environment. Migrating birds, fishes and whales, and even dogs walking with their owners, have different sensations, perceptions and a different “understanding” of the physical reality. As we mentioned in the introduction, in order to survive, they had to acquire a rudimentary notion of causality. Birds construct complex nests and follow sophisticated mating rituals; chimps and gorillas make strategic plans, construct simple tools and carry them to the place they need them to use.

We know now that a number of species such as gorillas, rhesus, capuchin, squirrel monkeys, lemurs, dolphins, elephants, black bears, birds, salamanders and fish developed numerical abilities. Even a 3-day-old domestic chicken differentiates between numbers [13]. When it sits in front of two small opaque screens and one ball disappears behind the first screen, followed by four balls disappearing behind a second screen, the chicken walks towards the screen that hides four balls. It is even more impressive that when two balls are moved from the second screen to the first screen, 80% of the time the chicken decides to walk to the first screen, “evaluating” that now there are more balls behind the first screen than behind the second screen. Chimpanzees are able to select quickly the set of bowls containing the largest combined number of chocolate pieces by adding together the number of pieces in each individual bowl [13].

A recent study by Martin Muller and Rudiger Wehner demonstrated that the Tunisian desert ants, in spite of the lack of visual landmarks and scent trails, are able to always compute their present location and to return to their nest by choosing the direct route rather than retracing its outbound trajectory [14]. This is why it would be surprising if the dinosaurs could not count.

Homo Sapiens developed quite early superior counting and reasoning skills. The first numbers were used in the Middle East around 10,000 BC. Counting started with the number one and evolved from using fingers and tally marks to sets of glyphs representing any conceivable number.

Babylonian mathematics is impressive [15]. They used accounting devices, such as bullae and tokens, as early as the 5th millennium BC. The majority of recovered clay tablets date from 1800 to 1600 BC, and cover topics that include fractions, algebra, quadratic and cubic equations and the Pythagorean Theorem.

Babylonians used a sexagesimal (base 60) numeral system because “60” has 10 different divisors, which is crucial in calculations with fractions. In comparison, “10” has only two divisors. Moreover, they were probably the first to use the positional notation, where digits written in the left column represented larger values. They also introduced written symbols for digits. We inherited from them the usage of 60, 360, 12 and 24.

The Babylonian clay tablet YBC 7289 (c. 1800–1600 BC) gives an approximation of √2 in a four sexagesimal figure, 1; 24, 51, 10, which is accurate to about six decimal digits [15], as follows:

(1) $$\sqrt{2}\approx 1;24,51,10=1+\frac{24}{60}+\frac{51}{60^{2}}+\frac{10}{60^{3}}=\frac{30547}{21600}\approx 1.41421\bar{2}\bar{9}\bar{6}$$

As well as arithmetical calculations, Babylonian mathematicians also developed methods of solving equations without using algebraic notation. These were based on pre-calculated tables. Babylonians measured perimeters, areas and volumes using the correct rules. For example, they used 3 or later 25/8 to approximate π. A circle’s perimeter was equal to three diameters and a circle’s area was equal to three radiuses squared. They knew and applied the Pythagorean rule. Babylonian astronomers kept detailed records of the rising and setting of stars, the motion of the planets, and the solar and lunar eclipses, all of which required familiarity with angular distances measured on the celestial sphere [15].

Egyptian mathematics developed from around 3000 BC to 300 BC [16]. The ancient Egyptians utilized a numeral system for counting and solving written mathematical problems, often involving multiplication and fractions. Egyptians understood quadratic equations and concepts of geometry, such as determining the surface area and volume of three-dimensional shapes, useful for architectural engineering.

**Figure 1:**
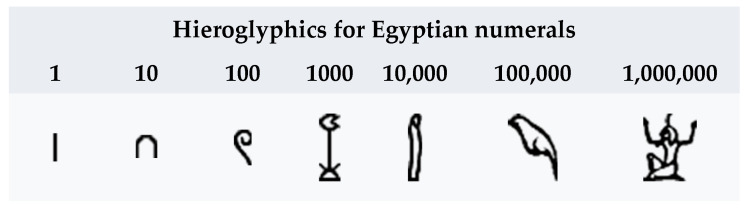
Hieroglyphics from Egyptian numerals. Complex numbers were formed by addition. For example, writing from right to left, 23 was depicted as 111∩∩.

Ancient Egyptian texts could be written on papyruses in either hieroglyphs or in hieratic. The number system was always given in the base 10. The number “1” was depicted by a simple stroke; the number “2” was represented by two strokes, etc. The numbers 10, 100, 1000, 10,000 and 100,000 had their own hieroglyphs. The number 1000 is represented by a lotus flower; the number 100,000 is represented by a frog, etc. (See Figure 1).

**Figure 2:**
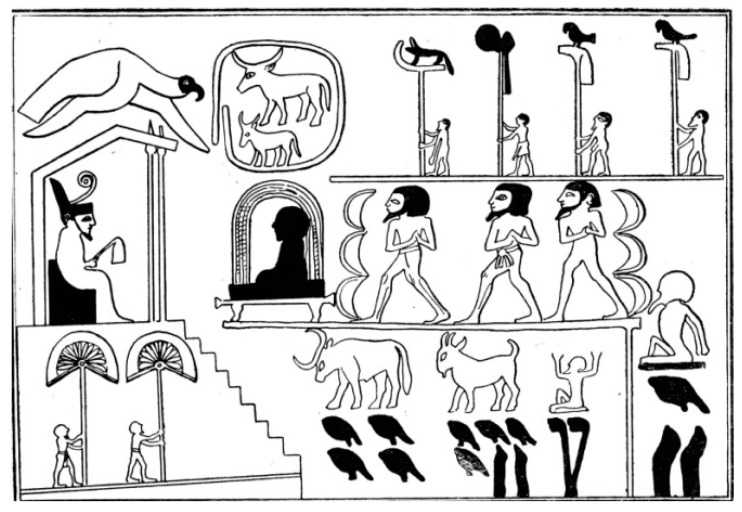
Glyphs copied from a decorated mace head, which depicts a ceremony where captives and other gifts are presented to Pharaoh Narmer, c. 3100 BC, who is enthroned beneath a canopy on a stepped platform.

The Egyptian number system was additive. Large numbers were represented by collections of the glyphs [16]. The impressive evidence of the use of the base 10 number system can be found on the Narmer Macehead [17], which depicts offerings of 400,000 oxen, 1,422,000 goats and 120,000 prisoners (See Figure 2).

**Figure 3:**
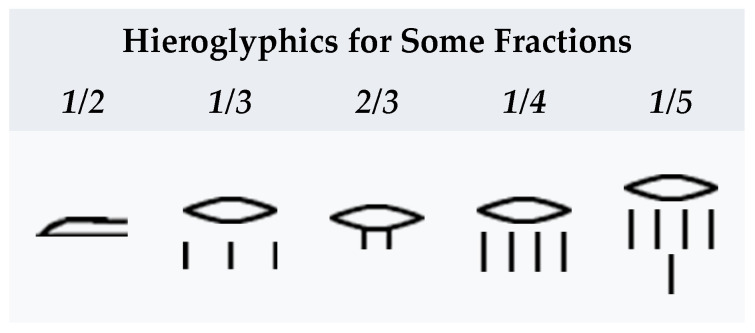
The fraction 1/2 was represented by a glyph that may have depicted a piece of linen folded in two. The fraction 2/3 was represented by the glyph for a mouth with 2 (different-sized) strokes. The rest of the fractions were always represented by a mouth superimposed over a number.

An interesting feature of ancient Egyptian mathematics is the use of unit fractions. With the exception of 1/2, 1/3 and 2/3, Egyptians used unit fractions in the form 1/*n* or sums of such unit fractions. Scribes used tables to rewrite any fraction as a sum of unit fractions [16] (See Figure 3).

Babylonians and Egyptians developed sophisticated mathematical tools to solve concrete and even complicated problems in everyday life, accounting and architecture. They were also able to predict seasonal changes and astronomical events. More information can be found, for example, in the excellent articles on Wikipedia [15,16,17].

## 3. From Pythagorean Pebbles to Euclidean Geometry and Ptolemy’s Model

Abstract concepts of numbers, geometrical figures and solids were created and studied extensively by the Greeks, who can be considered the fathers of modern mathematics, which became the indispensable tool for modeling physical reality.

Pythagoras was born on the island of Samos, and around 570 BC he settled in Croton, where he established the first Pythagorean community, described as a secret society [18]. Pythagoreans came up with an idea of numbers as symbols instead of just being numerals. They believed that whole numbers could explain the true nature of the universe. Numbers not only described important regularities and harmony in the world, but they also represented certain concepts and social relationships. Number one was identified with reason and being, two was identified with opinion, four represented justice, five signified marriage, seven was identified with health and eight with love and friendship [3,19,20].

**Figure 4:**
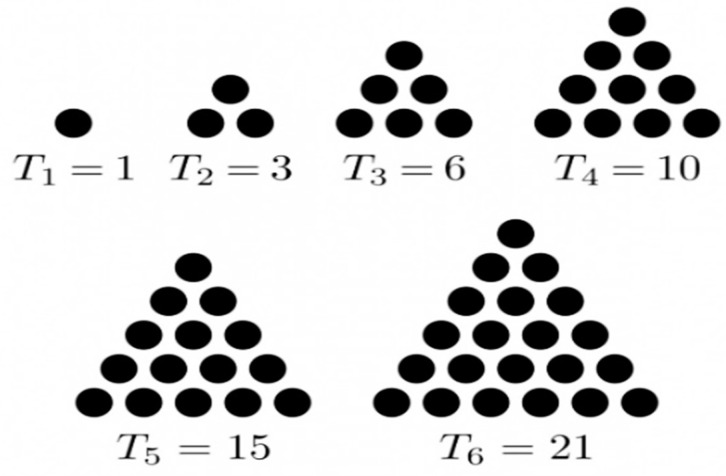
The first six triangular numbers.

Pythagoreans used pebbles to represent numbers in triangles, squares, rectangles and pentagons. This helped them to investigate the relationships between different numbers. They defined prime numbers, triangular, square, and odd and even numbers. Particularly important was the sacred number “10” (called Tetractys) because there were 4 pebbles on each edge (See Figure 4).

The geometrical representation of numbers allowed the detection of several regularities and the proof by inductions of several theorems. Since 1 + 3 = 4, 3 + 6 = 9, 6 + 10 = 16, thus any square number can be represented as a sum of two subsequent triangular numbers.

**Figure 5:**
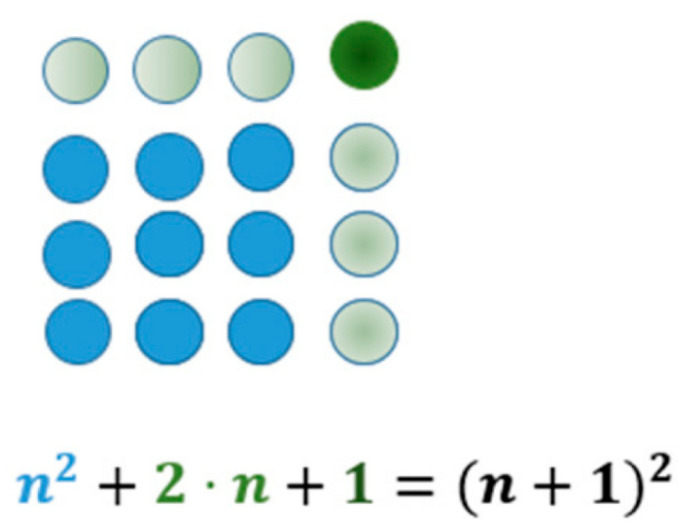
We easily notice that 3^2^ + 2 × 3 + 1 = 4^2^, etc. The number 2*n* + 1 was called gnomon.

Using Figure 5 we can derive another interesting theorem. We notice that 1 + 3 = 4, 1 + 3 + 5 = 9 and 1 + 3 + 5 + 7 = 16. We also see that 7 = 2 × 4 − 1 and 16 = 4^2^, thus by induction we conclude the following:

(2) $$1+3+\ldots +(2n-1)=n^{2}$$

which is valid for all *n* greater or equal to 1.

**Figure 6:**
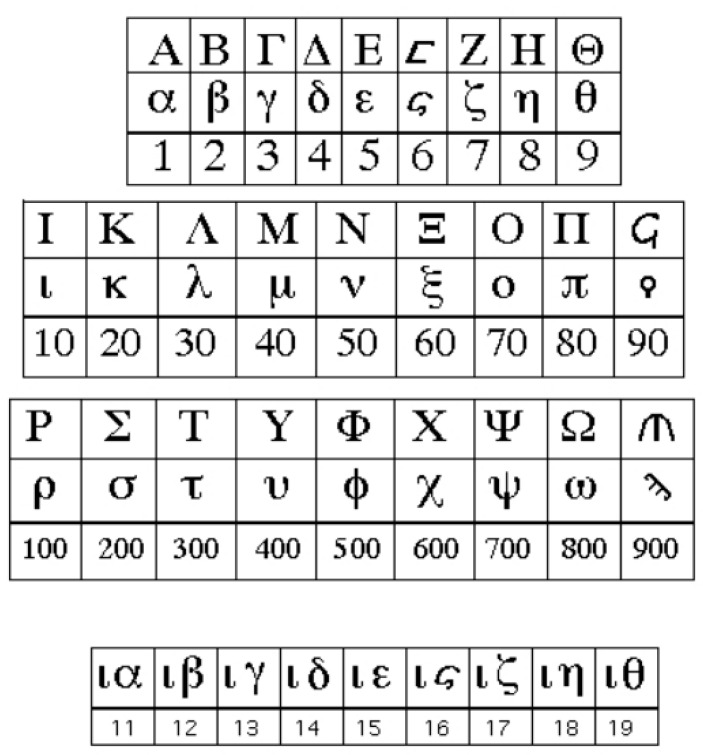
Greeks’ numbers represented by letters.

Pythagoreans defined a specific numerology believing that a person’s date of birth corresponds to a specific combination of numbers which can be used to describe their psychological type [19]. Moreover, they associated numbers with letters; this is why later Greeks in their manuscripts and books denoted numbers in combination with letters (See Figure 6).

They also searched for the perfect numbers as the sum of all their divisors, such as 6 = 1 + 2 + 3, 28 = 1 + 2 + 4 + 7 + 14. Since the next perfect numbers were 496, 8128 and 33,550,336, Nichomachus concluded, “*the good and beautiful are rare and easy counted, but the ugly and bad are prolific*”.

Pythagoreans discovered the Pythagorean Theorem and proved other simple geometrical theorems, including “*the sum of the angles of a triangle equals two right angles*”. They also studied three regular solids, the tetrahedron, the cube and the dodecahedron. They demonstrated that in the pentagram, each diagonal divides the two others at the golden ratio. When linear geometrical figures replaced the dots, the combination of Babylonian algebra with Pythagorean arithmetic provided the basis for Greek geometric algebra.

**Figure 7:**
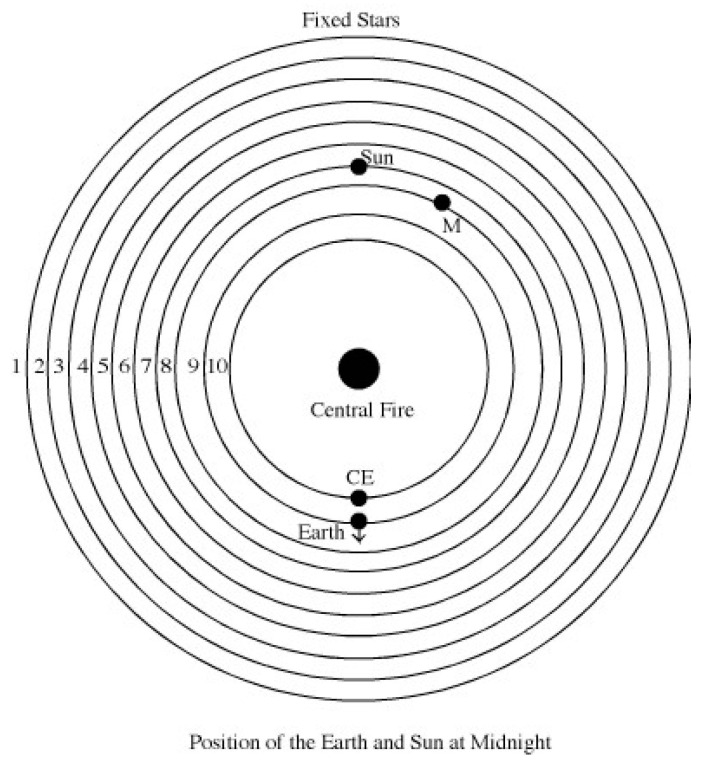
The incomplete diagram of the model of the universe proposed by Philolaus of Croton. We see only Central Fire, Sun Moon, Earth and CE (Anticthon–Counter Earth. Five more distant, known planets and the celestial sphere of stars are missing. The existence of Anticthon helped explain the diurnal cycle [22]. At midnight CE is blocking completely the light coming from the Sun.

Pythagoreans, Aristotle and Plato believed that numbers are the essence of matter, and that nature is composed of “*fourness*” [20,21]. The point, line, surface and solid are the only four possible dimensions of all forms. All matter is built out of four elements, earth, air, fire and water. Unlike most Greeks, they believed that the Earth is in motion and that there should be 10 celestial bodies because 10 was the sacred number [20]. Philolaus of Croton proposed the following model of the universe: the Earth, Moon, Sun, five remaining known planets, the sphere of the stars and Anticthon (invisible Counter-Earth) were revolving around a fixed central fire (See Figure 7).

Pythagoreans believed that the planets produced sounds which varied with their distances from the Earth and that all these sounds were harmonized. Nearly 2000 years later, Kepler, searching for harmony in the music of spheres, discovered his three important laws.

As “10” was a sacred umber, nature should be describable in terms of 10 pairs of categories such as, odd and even, bounded and unbounded, right and left, one and many, male and female, good an evil. The natural science of Pythagoreans was speculative and not satisfactory, but they recognized the importance of numbers underlying diverse natural phenomena.

Numbers and geometrical figures are suggested by physical objects, but Greek philosophers understood that they were abstract idealized concepts and undertook extensive study of their properties. These studies were resumed, extended and arranged by Euclid around 300 BC in The Elements, divided into 13 books. Starting from one set of 10 axioms, which seemed to be unquestionable, he rigorously deduced 467 interesting theorems and many corollaries. Axioms 1, 2, 3 and 5 assert the existence and uniqueness of certain geometric figures and Euclid explains how they can be constructed with no more than a compass and a straightedge.

Abstract geometry not only helped calculate distances and areas in everyday life, but due to the contributions of Archimedes, Aristarchus of Samos, Eratosthenes, Apollonius of Perga and Hipparchus, it led to the quite precise Ptolemaic geocentric planetary model [23], which survived 1500 years until the Copernican revolution. In fact, Aristarchus of Samos was the first to propose the heliocentric planetary system and perhaps his idea inspired Copernicus.

The Ptolemaic system provided an accurate predictive model for celestial motion. In this system, Earth is stationary and at the center of the universe. Heavenly bodies move in uniform motion along the most “perfect” path, which was considered to be a circle. To explain the apparently irregular movements of planets, seen from the perspective of a stationary Earth, Ptolemy assumed that they were a combination of several regular circular motions called epicycles. Specifically, each planet revolves uniformly along a circular path called an epicycle, and the center of the epicycle itself revolves around Earth along a larger circular path called the deferent. Ptolemy had to place the Earth not at the center of the deferent, which was called eccentricity, to explain the varying motion of the Sun through the zodiac.

The beauty and rigor of deductive reasoning in Euclid’s Elements has been inspiring philosophers and scientists until today. Being an obligatory subject taught in schools for several centuries, it contributed significantly to the success of the technological and scientific revolution initiated by Galileo, Copernicus and Kepler in the 16th century.

As we mentioned above, Pythagoreans recognized the importance of numbers, in particular whole numbers, and they made the first steps toward applying this concept to the study of nature. Over the centuries, the concept of numbers has been extended and efficient schemes of writing them and calculating with them have been created. Zero, negative numbers and negative decimal fractions were defined, but only in the 17th century, mathematicians generally tend to use them in modern notation. Irrational numbers and negative numbers were often considered to be absurd and even Descartes rejected negative solutions of algebraic equations.

Only in the 19th century did mathematicians accept complex numbers, separated irrationals into algebraic and transcendental parts and undertook the serious scientific study of irrationals, a topic which remained almost dormant since Euclid. More information about the history of numbers may be found in [3,24,25].

It is impressive that the uses of numbers we follow today for understanding and mastering our description of nature are similar to those made by Pythagoreans. As Kronecker said, “God created the integers, all else is the work of man”. We will talk about this in the subsequent sections.

## 4. Copernican Revolution and Newtonian Classical Mechanics

Following the fall of Rome, monasteries and convents remained bastions of scholarship in Western Europe, and clergymen were the leading scholars of the age—studying nature, mathematics, and the motion of the stars (largely for religious purposes) [26]. The Council of Nicaea prescribed that Easter would fall on the first Sunday following the first full moon after the vernal equinox. Thus, it became necessary to predict the date of Easter with enough accuracy. This necessity fueled the constant innovation and refinement of astronomical practice as the solar and lunar years diverge over centuries. In the 12th century, the church sponsored the translation into Latin of Arabic-language versions of Greek philosophical and mathematical texts. This was achieved to help astronomical study.

**Figure 8:**
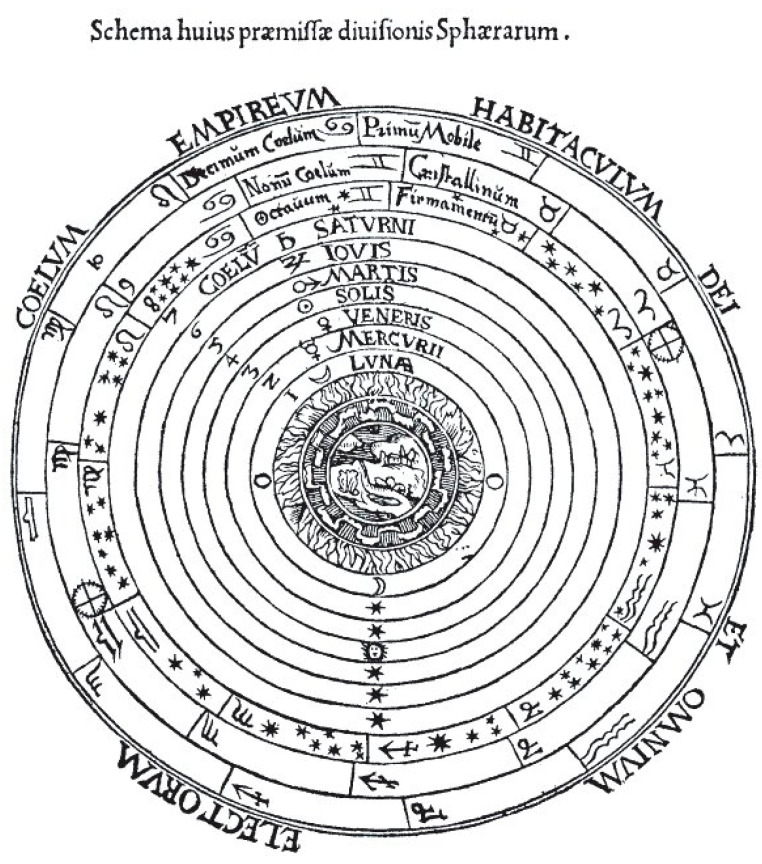
Early printed version of Ptolemaic system (Christian Aristotelian cosmos. From Peter Apian, Cosmographia, 1524. Earth is in the center and Sun (Solis) is in between Venus and Mars.

Aristotle put Earth in the center of the cosmos and the Ptolemaic geocentric model seemed to reinforce the message of creation in the Bible and other Sacred Scriptures (See Figure 8).

**Figure 9:**
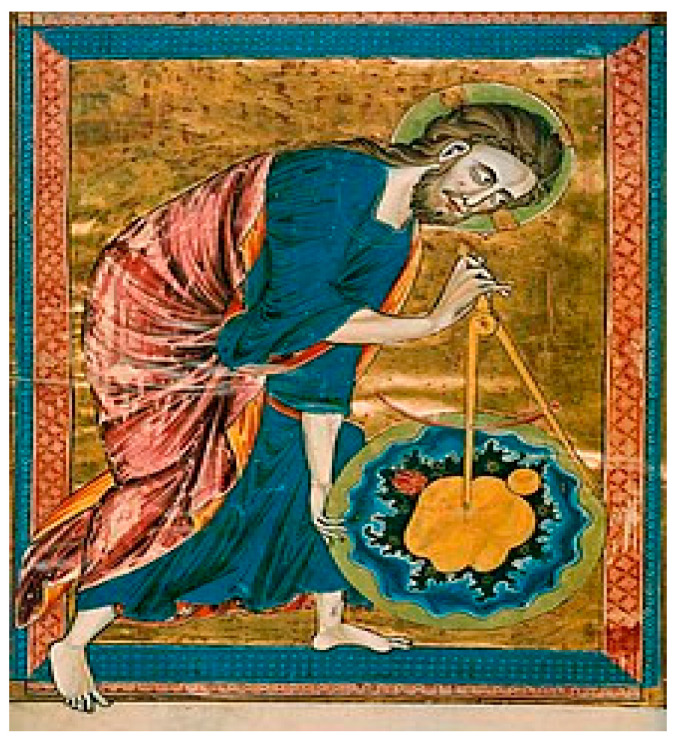
God the Geometer—Gothic frontispiece of the Bible moralized, representing God’s act of Creation. France, mid-13th century.

The Catholic Church has been an important patron of the sciences, arts and architecture. It played a significant role in the foundation and funding of schools and hospitals. Some cathedral schools became the first universities. Catholic scientists, both religious and lay, have led scientific discovery in many fields, searching for the divine design of the world, which might be considered as additional proof of the existence of God [26] (See Figure 9).

The Church also tolerated Aristotelian science, which was taught and venerated by scholars in universities. Aristotle’s cosmos was a series of concentric spheres. The terrestrial sphere was composed of four elements, earth, air, fire, and water. These elements were subject to change and decay. The celestial spheres were made of unchangeable aether. Aristotle explained phenomena on Earth in terms of qualities or substances, e.g., hot and cold, wet and dry, solid and fluid, etc. Objects made of earth and water tended to fall and the speed of motion depended on their weights and the density of the medium. To maintain the constant motion of the body, the force has to be constantly applied. The objects made from air and fire tended to rise. The vacuum could not exist because speeds would become infinite. Aristotle insisted on the causal explanation of any changes and defined them as material, formal, efficient and final causes.

The conflict between the Church and science started when Nicolaus Copernicus constructed a precise heliocentric model of the planetary system in the book De Revolutionibus…, published in 1543. According to this model, Earth lost its privileged place in the universe. It was revolving around the Sun, like other planets, and it was rotating around its axes. At the beginning, in realizing that the Copernican model allowed more precise astronomical predictions, the Church considered it to be false but useful and did not declare it as heresy.

Copernicus’ theory lacked the necessary evidence to be universally accepted. There were several unanswered questions, such as how a heavy object like Earth can be kept in motion or why the Earth’s rotation does not cause objects to fly away, thus the Copernican model was only a bold but questionable hypothesis. Nevertheless, when Galileo in his book, Dialogue Concerning the Two Chief World Systems, explicitly endorsed the Copernican model, breaking the agreement with Pope Urban VIII, he was forced to recant and was sentenced by the inquisition to house arrest. The Copernican model was declared a dangerous heresy, contrary to Holy Scriptures. De Revolutionibus and Galileo’s Dialogue Concerning the Two Chief World Systems were only dropped from the Catholic Church Index of Prohibited Books in 1835 [3].

For Galileo, faith and reason were complementary, this is why he endorsed and promoted the Copernican heliocentric model. He demonstrated that several Aristotelian views were wrong. He pointed out that one should not describe nature by qualities such as white or red and sound or silence but by measurable observables like shape, quantity and motion. He formalized the concept of experimentation and recording results. Using the lever law, he could measure the specific gravity of objects by immersing them in water and balancing weights. He used a telescope to observe Jupiter’s moons, sunspots, the phases of Venus and challenged the idea of a perfect celestial sphere. He disproved Aristotelian dynamics and discovered that a falling object accelerated at the same rate regardless of its weight (in the absence of air resistance). He also showed that projectiles follow a parabolic path. His work on inertia contributed to the formulation of the Newton’s first law.

Kepler improved the Copernican heliocentric system and discovered the following three fundamental laws that describe how planets move around the Sun:

1. Planets move in elliptical orbits, with the Sun as one of the foci.
2. A line joining the Sun and a planet sweeps out equal areas in equal times.
3. There is a precise relationship between a planet’s orbital period and its average distance from the Sun.

Kepler and Copernicus asked man to accept a theory that violated his senses’ impressions because this is a more satisfactory mathematical theory. They believed that reason and mathematics should be the determining factor in accepting what is true in nature [3]. Modern science follows this line of thought.

Reason and mathematics were also the fundamental methods of inquiry recommended by René Descartes. He said that in order to search for truth, it was necessary, once in the course of one’s life, to doubt all things. In Discourse on Method, he constructed his philosophy by a deductive method based on the axioms that seemed self-evident to him.

In his Geometry, he connected the previously separate fields of geometry and algebra, creating analytical geometry. The Cartesian coordinate system, which we commonly use today, was named after him. In this system, geometric points on the plane are uniquely specified by a pair of real numbers (coordinates) representing their distances from two fixed perpendicular lines (the coordinate axes). For the points in space, one has to add an additional coordinate axis. Descartes demonstrated that to each curve there belongs an equation that describes the position of any point on the curve. Moreover, each equation relating to x and y can be pictured as a curve on a plane. In this way, all paths, curves and surfaces that occur in the physical world can be studied efficiently using algebraic methods.

Newton’s contributions to mathematics and physics were vast, including his development of calculus, laws of motion, and universal gravitation. Newtonian mechanics describes the motion of objects based on deterministic laws. If we know the initial conditions (positions and velocities) of all objects in the universe and the forces acting upon them, we can precisely predict their future behavior. The three Newtonian law of motions and the 4th law of universal gravitation laid the foundation for classical physics, which remains valid for most everyday scenarios.

Newton’s three fundamental laws of motion are as follows:

1. An object at rest remains at rest, and an object in motion continues moving with constant velocity unless acted upon by an external force.
2. The acceleration of an object is directly proportional to the net force applied to it and inversely proportional to its mass (F = ma).
3. For every action, there is an equal and opposite reaction.
4. Every mass attracts every other mass with a force proportional to the product of their masses and inversely proportional to the square of the distance between them.

Newton introduced an important notion of a mass point as an idealization of material bodies which are far away. This allowed him to derive the motion of planets, consistent with the heliocentric system and with Kepler’s laws. The gravitation force is defined between any two mass points, and if there are many mass points, the force acting on a particular mass point is the sum of all the forces acting on it. Newton knew that planets are not points but spheres. However, massive solids can be described as rigidly connected material points or by assuming a continuous mass distribution and defining the mass density. This is probably why Newton waited for 20 years before publishing his Mathematical Principles of Natural Philosophy only in 1687, when he demonstrated that the gravitational force between two spheres can be calculated as their total masses located in their centers.

Using his law of gravitation, he calculated the masses of the sun and all the planets, explained ocean tides, etc. His Principia inspired and guided subsequent generations of scientists. In the preface to the first edition, he defined a program of research which did not lose its validity today, “I offer this work as mathematics principles of philosophy [science]; for all the difficulty in philosophy seems to consists in this–from the phenomena of motions to investigate the forces of nature, and then from these forces to demonstrate other phenomena”.

Newton’s law of gravitation asserts that the force of gravitation acts between the Sun and planets over huge distances. This was in conflict with general beliefs because, as Aristotle said, “action and passion (in the proper sense of the terms) can only occur between things which are such as to touch one another”. The gravitational force was exerted locally on each planet, but it acted instantaneously and constantly through empty space, and it could not be blocked. This is why Newton wrote: “I here design only to give mathematical notion of these forces, without considering their physical causes and seats”.

In Einsteinian theory of gravity, which is another abstract mathematical model, one is not talking about the forces. Objects move along the geodesics in a 4-dimensional curved space-time. The curvature represents gravity and depends on the relative positions of massive objects. When a planet orbits the Sun, it is essentially following the geodesic determined by the Sun’s mass and the curvature of space-time. In general relativity, the light follows different geodesics and massive objects (like galaxies) bend light as it passes near them. This effect, called gravitational lensing, has been observed and confirmed. General relativity similarly to Newtonian mechanics does not answer a question “Why” but a question “How”. We do not know the physical causes and saying that massive objects warp the fabric of space-time around them as a heavy ball on a trampoline is simply misleading. Both Newtonian and Einsteinian theories are only mathematical abstract models of some aspects of physical reality.

Standing on the shoulder of the giants, Copernicus, Kepler and Galileo, Newton provided a comprehensive, systematic and rationally connected account of terrestrial and celestial motions. He established the existence of universal mathematical laws, providing strong arguments in favor of the mathematical design of the universe. This allowed sweeping away the last traces of mysticism [3].

Over the next 200 years, Newtonian mechanics has been inspiration for philosophers, physicists and mathematicians. Newton’s laws were used to describe solids, liquids and gasses. In order to solve complicated physical problems, new mathematical concepts and methods were defined and studied, such as ordinary differential equations, partial differential equations, integral equations and a calculus of variations. One may say that it was a golden epoch of science due to the continuous “cross-fertilization” between physics and mathematics. In fact, Euler, Lagrange, d’Alambert, Bernoulli, Laplace, Hamilton and several other scientists made equally important contributions to physics and mathematics.

Newton’s equations of motion, in contrast to the average velocity, contained instantaneous velocity and acceleration. A position of the body at time *t* in a chosen Cartesian reference frame is described by a vector **r**(t) = (x(t), y(t), z(t)) and instantaneous velocities and accelerations are defined as follows:

(3) $$v(t)=\dot{r}(t)=\lim_{h\to 0}\frac{\left(r(t+h)-r(t)\right)}{h};\;a(t)=\ddot{r}(t)=\dot{v}(t)$$

In Equation (3) and in equations which follow I could not incorporate bold characters, thus the vector functions **r**(t) is denoted *r*(*t*), ***v*(***t*) is denoted ***v*(***t*) etc. If the initial position ***r***(t_0_) and the velocity ***v***(t_0_) are known, the future motion of a material point of mass *m*, in the absence of constraints is strictly predetermined by Newton’s second order differential equation:

(4) $$m\ddot{r}=F(r,\dot{r},t)$$

where $F(r,\dot{r},t)$ are the external forces acting on a mass point. Equation (3) is a vector notation for a system of three differential equations of the second order for functions x(t), y(t) and z(t). For one mass point, the most important forces are: **F** = m**g** and central forces **F**(**r**) = f(|**r**|)**r,** where |**r**| is the length of the vector **r**, in particular if f(|**r**|)= k(|**r**| and f(|**r**|)= c/|**r**|^−3^ (where k and c are some constants). The work exerted to move a material point in the field of central forces from point P to point Q does not depend on the path. The total angular momentum and total energy, being a sum of kinetic and potential energy, are conserved.

To describe motions of N material points in the presence of constraints, physicists had to introduce generalized coordinates and solve complicated differential equations. Often, there were no exact solutions and only approximate solutions could be found. The equations of motion are nowadays derived using the Least (Stationary) Action Principle [3,27,28,29,30], which also plays a fundamental role in quantum electrodynamics and in quantum field theory. We briefly discuss this principle and the development of Hamiltonian mechanics in Appendix A.

The Least Action Principle can be generalized for various physical systems including electromagnetism, relativity, and quantum mechanics. Its importance cannot be underestimated because Noether’s theorem [31] connects symmetries to conservation laws.

1. Translation Symmetry: If the action is invariant under translations in space (i.e., the laws of physics remain the same regardless of where we are in space), then the linear momentum is conserved.
2. Time Translation Symmetry: If the action is invariant under translations in time (i.e., the laws of physics remain the same regardless of when we observe them), then energy is conserved.
3. Space Rotation Symmetry: If the action is invariant under rotations in space, then the angular momentum is conserved.

Symmetry transformations play a crucial role in understanding the fundamental laws of physics. In particle physics, several additional intrinsic discrete symmetries and the corresponding conservation laws were discovered and helped physicists to construct the Standard Model [32,33].

It was difficult and practically impossible to find the solutions of Newton’s equations for a system of many material points, but it was believed that, if one knew the general solution, initial positions and velocities of all these points, then the future evolution of the universe could be predicted. As we explain in the next section, this belief is unfounded.

## 5. Three-Body-Problem, Strange Attractors and the Chaos Theory

Newtonian mechanics is a deterministic theory, and if we know the initial conditions, the future of a physical system is completely determined. However, Newton’s equations are difficult to solve if the number of material points is increasing. This is why, in 1887, Oscar II, king of Sweden, established a prize for anyone who could find the solution to the *n*-body problem:

Given a system of many arbitrary mass points that attract each according to Newton’s law, under the assumption that no two points ever collide, trying to find a representation of the coordinates of each point as a series in a variable that is some known function of time and for all of its values, the series converges uniformly.

In 1881–1882, Henri Poincaré showed that it is possible to derive the important information about the behavior of a family of solutions of the differential equations without having to solve the equation (since this may not always be possible). He successfully used this approach to prove that there is no solution to the *n*-body problem and that even the deterministic system of three bodies can exhibit chaotic behavior, strongly dependent of the initial conditions [34,35,36].

**Figure 10:**
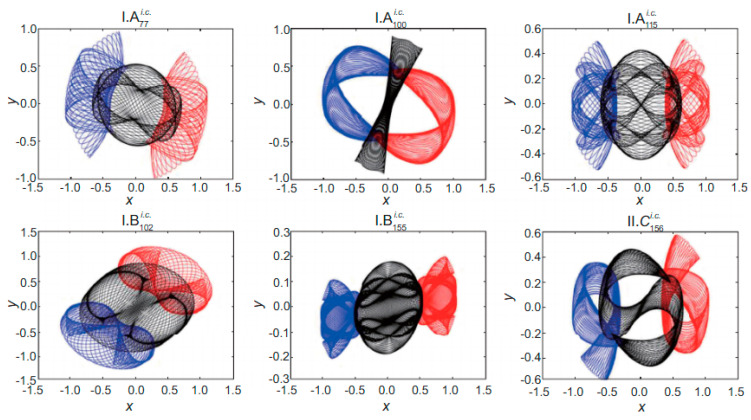
Six families of periodic orbits discovered recently by two Chinese scientists.

The Three-Body-Problem (TBP) is a system of nine differential second order equations describing the possible motions of three point masses which attract each other through gravity. A general solution to these equations does not exist. The motion of the three bodies is generally chaotic for most initial conditions. Only if the mass of one body is much smaller than the other two masses one may find analytic solutions. Therefore, to determine how the positions change in time, computer simulations have to be used. In 2017, two scientists, XiaoMing Li and ShiJun Liao, using a supercomputer, determined 695 families of periodic orbits of planar TBP [37,38]. In their simulation, the gravitational constant G = 1, all masses are equal to 1 and are placed in the corners of the isosceles triangle (See Figure 10).

**Figure 11:**
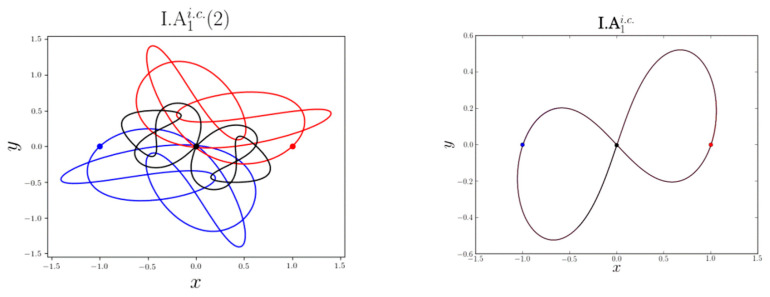
Two examples of periodic orbits for equal masses.

Detailed characteristic parameters (such as periods, scale-invariant averaged periods, initial velocities, etc**.**), and the motions on these periodic orbits can be found in [38] (See Figure 11).

**Figure 12:**
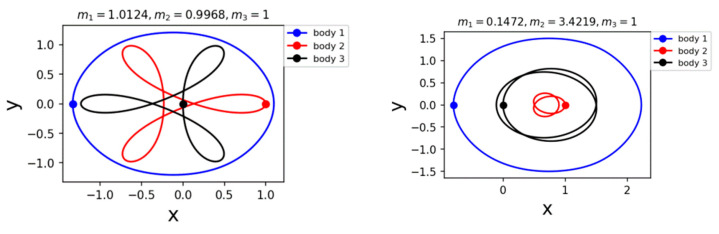
The relatively periodic BHH satellites orbit the three-body system with various masses in a rotating frame of reference. Blue line: body-1; red line: body-2; black line: body-3.

In their subsequent publications, they also found several periodic and chaotic families for non-equal masses [39,40,41] (See Figure 12).

TBP is inherently chaotic. No computer can predict the behavior of three bodies indefinitely for all possible initial conditions and chosen values of the three masses. The orbits become unpredictable, leading sometimes to cataclysmic events such as collisions or one planet leaving the system. Nevertheless, the computer simulation allows us to discover some regular patterns such as periodic orbits and attractors. Attractors are sets of points to which a system tends to evolve regardless of its initial conditions. A strange attractor is a specific type of attractor characterized by a sensitive dependence on initial conditions.

A strange attractor is a set of points in phase space (the space of all possible system states) that describes how a chaotic system evolves. We cannot precisely predict where on the attractor the system will be at a given time. Small differences in initial conditions lead to vastly different trajectories on the attractor. Strange attractors have intricate shapes and are often characterized by fractal-like patterns.

**Figure 13:**
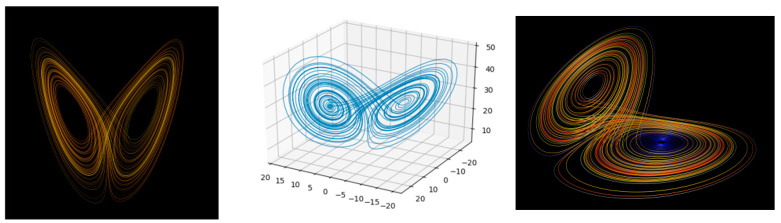
Lorentz strange attractor and the butterfly effect.

A classic example is the Lorenz attractor, better known as the “butterfly effect” image. Edward Lorenz and collaborators used a set of three simple equations to model the Earth’s dry atmospheric convection and noticed that no reliable predictions could be made about the future behavior of this deterministic system [42]. Nevertheless, some regularities were observed, and the possible motions of the system were limited to some region of space, which is now call the Lorenz attractor [43] (See Figure 13).

The discovery of the chaotic behavior in TBP and Lorentz attractor contributed to the creation of the chaos theory, which is an interdisciplinary branch of science and mathematics studying deterministic systems which are predictable for a while and then ‘appear’ to become random. Examples of chaotic systems include a double-rod pendulum, fluid dynamics, climate and weather processes, biological processes, heart arrhythmias population dynamics and stock markets valuations [44].

The amount of time for which the behavior of a chaotic system can be effectively predicted depends on the following three conditions: how much uncertainty can be tolerated in the forecast, how accurately its current state can be measured and what is a time scale, called Lyapunov time, characterizing the dynamics of the system. Lyapunov time for chaotic electrical circuits is about 1 millisecond; for weather systems a few days and for the inner solar system 4 to 5 million years.

In chaotic systems, the uncertainty in a forecast increases exponentially with elapsed time. This means, in practice, that a meaningful prediction cannot be made over an interval of more than two or three times the Lyapunov time. Since the Lyapunov time for the inner solar system is very long, the orbits of Earth and other close planets will remain stable in the human time scale.

Within the apparent randomness of chaotic complex systems there are underlying patterns, repetition, self-similarity, fractals and self-organization. The “butterfly effect”, an underlying principle of chaos, describes how a small change in one state of a deterministic nonlinear system can result in large differences in a later state [45,46]. A metaphor for this behavior is that a butterfly flapping its wings in Brazil can cause a tornado in Texas.

Chaos theory has become applicable to geology, mathematics, biology, computer science, economics, engineering, finance, meteorology, philosophy, anthropology, physics, politics, population dynamics and robotics.

As we mentioned above, strange attractors sometimes have fractal structures. Fractals are mathematical objects characterized by self-similarity patterns that reoccur at smaller and smaller scales [47]. We are going to discuss them in the subsequent section.

## 6. The Fractal Geometry of Nature

The term “fractal” was popularized by Benoit Mandelbrot in the 1960s and 1970s and has been studied intensively since [47,48,49,50,51].

The solutions of differential equations are smooth curves or surfaces, which means that a tangent line or a tangent plane exists at all points. In nature, we observe “roughness” (no tangent lines or planes do exist), thus, in order to describe this “roughness” and self-similar patterns we have to use different mathematical concepts and description than Newtonian mechanics.

As Mandelbrot said: “Clouds are not spheres, mountains are not cones, coastlines are not circles, and bark is not smooth, nor does lightning travel in a straight line” [51,52].

Long time ago, British cartographers encountered a problem in measuring the length of Britain’s coast. The coastline measured on a large-scale map was approximately half the length of coastline measured on a detailed map. It is obvious that the measurements of the length depend on the precision (the size and units of the measuring rod). However, if the curve is smooth, the measurements made with higher and higher precision converge to a constant value. If we have a rough object like a coastline, the measurements seem to diverge instead of converging. For fractals, the Euclidean measure tends to infinity, thus mathematicians and Mandelbrot decided to characterize the fractals by their fractional dimension D, which is consistent with the much more rigorously defined Hausdorff dimension.

**Figure 14:**
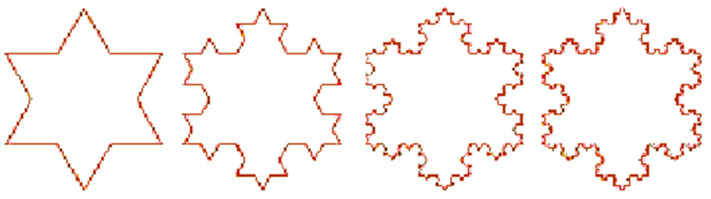
First 4 iterations of the algorithm constructing the Koch snowflake curve.

The dimension represents the measure of object changes, if we scale the unit of length. For example, let us start with a linear segment of the length 1. If we divide this length by S = 2 (scaling factor), we have N = 2 line segments of length ½ and N × (1/S)^1^ = 1. If we have a square 1 × 1, the measure on a plane is not the length but the area. Thus, if we divide each side by S = 2, then we obtain N = 4 small squares, each having the area ¼ and now N × (1/S)^2^ = 1. If we subdivide a unit cube into 8 small identical cubes, then measurements in space are the volume and also N × (1/S)^3^ = 1. By generalizing this approach, the Hausdorff dimension of the fractal may be defined as:

(5) $$N{\left(\frac{1}{S}\right)}^{D}=1\to D=\frac{\log N}{\log S}$$

where N is a number of self-similar pieces on which a geometric object is transformed after the first iteration and S is a scaling factor. As an example, we will calculate the fractal dimension of the Koch snowflake curve [52,53] (See Figure 14):

To construct the Koch snowflake, we start with an isosceles triangle with sides of length 1.

1. We divide each side into three equal segments of length 1/3
2. On the segment of each side, we add a new equilateral triangle one-third of the size, and we erase its base, thus, each side is replaced by four identical shorter segments.
3. We repeat this process to infinity.

The scaling factor S =3 and N = 4, thus the fractal dimension is $D=\frac{\log4}{\log3}\approx 1.26186$.

**Figure 15:**
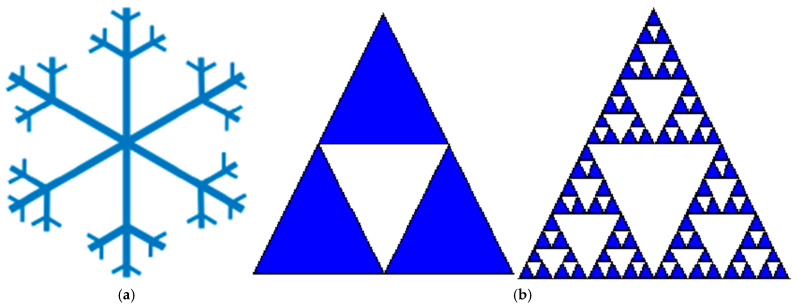
(**a**) Snowflake dendrite [53]; (**b**) the first and the fourth iteration of the Sierpinski gasket [54].

The dimensions of other fractals can be also easily calculated following Figure 15:

The dimension for (a) is $D=\frac{\log5}{\log3}$ and for (b) is $D=\frac{\log3}{\log2}$. As Pythagoreans anticipated, a few first natural numbers are important in nature.

Let us now calculate the length of the perimeter of the Koch snowflake. At each iteration, the length of each side is increased by a factor 4/3, thus after *n* iterations the perimeter P*_n_* = 3(4/3)*^n^* and tends to infinity when *n* increases. At the same time, the area remains smaller than the area of a circle drawn around the original triangle. That means that the infinitely long line surrounds a finite area. Similarly, the surface of the fractal surface around the finite volume may also be infinite. Koch’s snowflake resembles the coastline of a shore.

Various fractals can be constructed using similar algorithms. One can also construct higher dimensional fractals such as “rough “(nowhere smooth) surfaces having an infinite area around the finite volume. The fractal dimension is a measure of the space-filling ability of curves and surfaces with irregular shapes. For irregular surfaces, one covers their shades by a grid of squares and studies how the number N of squares intersecting the boundary of the shade changes when the scaling factor S changes. Next, for multiple values of S, one plots N vs. S as points on a log–log graph. The approximate fractal dimension of the boundary D_b_ is the slope of the best fit straight line through the points, and the approximate fractal dimension of the surface is D = D_b_ + 1 > 2.

**Figure 16:**
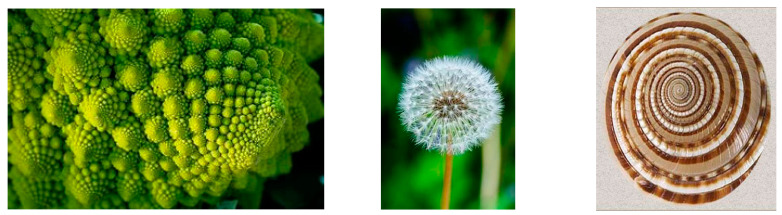
Three examples of fractal structures in nature.

**Figure 17:**
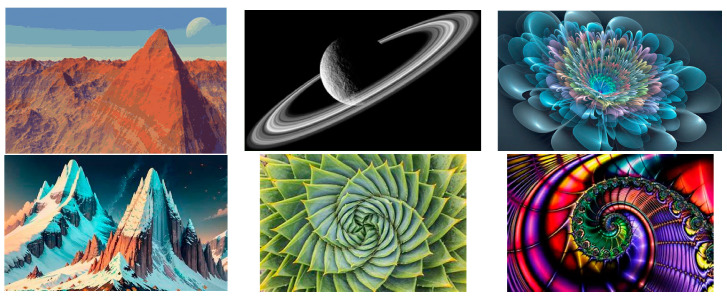
Fractal art inspired by nature. Colors at different points depend on how these points are transformed in successive iterations. Of course, the final choice is motivated by the artistic effect one wants obtain [51,52].

Many fractal patterns are found in nature, such as the following Figure 16 and Figure 17:

Koch snowflakes and the Sierpinski gasket are examples of the so-called Iterated Function System Fractals (IFS), created by iterating simple plane transformations, such as scaling, dislocation and plane axes rotation. Each point on the plane can be represented by a complex number z = x + iy. Displacements of points on the plane can be described by subsequent iterations of complex value functions, defined by the recurrence equation, $Z_{n}=f(Z_{n-1)}$.

To construct the Mandelbrot set M [47,52,55], we choose a constant complex number c, Z_0_ = 0 and a second order polynomial function as follows:

(6) $$Z_{n}={Z^{2}}_{n-1}+c$$

M is defined as a set of all complex numbers c, such that the sequences of points generated by repeatedly applying the quadratic map (6), called orbits, remain bounded. M is a compact single connected fractal set, since it is closed and contained in the closed disk of radius 2 around the origin.

1. A point inside M remains inside this set in all iterations of the map (6).
2. Points far from M rapidly move towards infinity.
3. Points close to M slowly escape to infinity.

**Figure 18:**
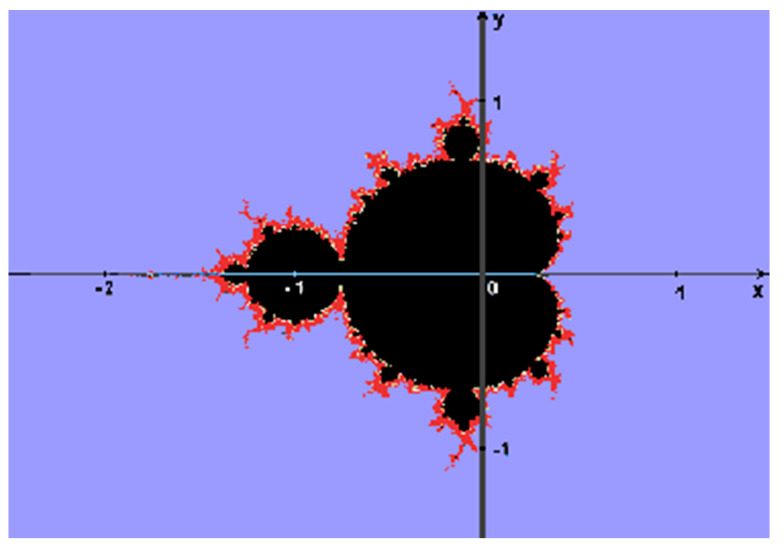
Mandelbrot set. A system in a black initial point remains inside the set. Colors indicate how fast a system in these points escapes to infinity.

**Figure 19:**
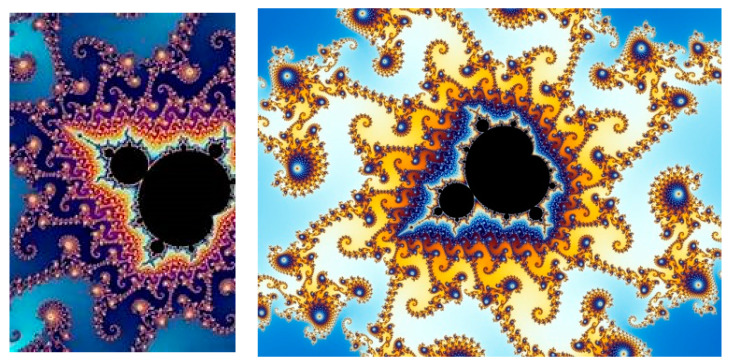
Details of the Mandelbrot set.

M may be depicted as a colorful image, where each pixel corresponds to a complex number, and its color depends on how many iterations were required to determine that they are outside the Mandelbrot set (See Figure 18 and Figure 19):

Another important fractal family are the *Julia sets* [47,52,56]. A Julia set, associated with a specific polynomial map, is the set of initial points, whose orbits exhibit certain behavior, where an orbit is a sequence of points generated by repeatedly applying the map to an initial point. If the orbit remains bounded, the point belongs to the filled Julia set. If the orbit escapes to infinity, the point belongs to the *basin of infinity*.

The Julia sets for the quadratic complex maps (6) are closely related to the Mandelbrot set, but now c is treated as the constant complex parameter, and for each c, we have a different uniquely filled Julia set of all the points satisfying the specific criteria. The quadratic complex map is defined as in (6) by the function *f_c_*(*z*) = *z*^2^ + *c*.

The filled Julia set for *f_c_*(*z*) is constructed as follows:

1. We choose the initial point z_0_ = x + iy from a rectangular grid on the complex plane such that {(x,y)|a≤x≤b,c≤y≤d}.
2. If the magnitude of z_n_ exceeds 2, we say that z_n_ escapes to infinity. Otherwise, we continue iterating until either the escape criterion is met, or a maximum number of iterations is reached.
3. If z_0_ escapes, its color is based on the number of iterations before escape (this creates intricate patterns). If z_0_ remains bounded, its color is usually black.
4. We repeat this process for all points in the grid.

**Figure 20:**
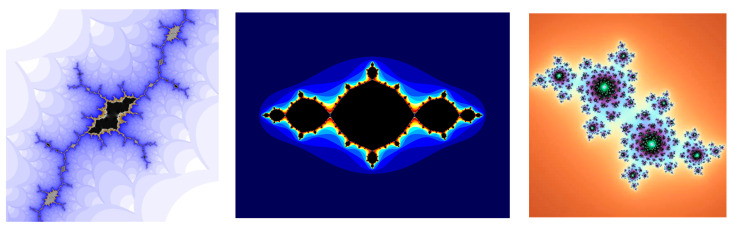
Connected and disconnected Julia sets.

The parameter space for a Julia set is the whole complex plane. In general, Julia sets are disconnected, and when c in the parameter space passes by the boundary, the Julia set changes abruptly and becomes connected. The phenomena by which smooth changes made to the parameter values (the bifurcation points) cause a sudden “qualitative” or topological change in its behavior are studied by the Catastrophe theory, created by Rene Thom. Using this definition, the boundary of the Mandelbrot set can be defined as the bifurcation locus of this quadratic family of mappings (See Figure 20):

Catastrophe theory [57,58] is a part of bifurcation theory, which studies and classifies phenomena characterized by sudden shifts in behavior due to small changes in circumstances. It analyzes the degenerate critical points of a potential function. For some values of certain parameters describing a nonlinear system, called bifurcation points, equilibria can appear or disappear, leading to large and sudden changes in system behavior. Catastrophe theory has been applied to various fields, including physics, biology, and social sciences. It can help explain phenomena like earthquakes, phase transitions, and biological shifts.

Chaos theory studies the behavior of dynamic systems that are highly sensitive to initial conditions. These systems exhibit unpredictable and complex behaviors, even though their underlying rules are deterministic. Bifurcations play a crucial role in chaos theory, as they lead to chaotic behavior [59,60]. Fractals are geometric shapes that exhibit self-similarity at different scales. As we saw, fractals are found in nature (coastlines, clouds, snowflakes) and are essential in chaos theory because they represent complex, infinitely detailed structures.

In summary, chaos theory, catastrophe theory, bifurcations, and fractals all contribute to our understanding of complex systems, their behavior, and the underlying mathematical principles. They reveal the beauty and intricacy of natural phenomena, from weather patterns to seashells.

They are sophisticated tools to model, often in a qualitative way, complicated nonlinear phenomena observed in nature, which cannot be described quantitatively by Newtonian mechanics.

## 7. From Democritus and Mendeleev

In this section, we resume the discussion of how a belief in the existence of quantitative laws of nature led scientists to sophisticated mathematical descriptions of various levels of physical reality, consistent with numerous experimental data.

The Greeks not only developed the abstract concept of numbers and geometry. Already around 400 BC, Democritus created the first atomistic theory, which after being criticized by Aristotle, was rediscovered after the Copernican revolution and led to the development of the modern atomistic theory. Probably inspired by Pythagorean pebbles and numerology, Democritus believed that all matter is made up of tiny, indivisible particles called atoms. Atoms varied in size, shape and weight. They were constantly in motion and could combine to form different substances. He believed that atoms are unchangeable and eternal, which was only disproved in the last 200 years.

The creation of Newtonian mechanics, the discovery of electromagnetic phenomena and electric currents was followed by the development of modern chemistry.

The history of chemistry reflects humanity’s quest to understand the composition of matter and its transformations, from ancient fire-making to cutting-edge scientific discoveries. Gold, silver, copper, tin and meteoric iron were among the earliest metals used by humans. The Varna culture in Bulgaria (around 4600 BC) practiced gold metallurgy.

As astrology led to modern astronomy, alchemy, which emerged during the Middle Ages, laid the groundwork for modern chemistry. Alchemists sought to transform base metals into gold and discover the elixir of life. The 17th and 18th centuries marked the transition from alchemy to modern chemistry. Scientists like Robert Boyle, Antoine Lavoisier and Joseph Priestley made significant contributions [61,62].

Antoine Lavoisier established the law of conservation of mass during chemical reactions. He also coauthored the modern system for naming chemical substances, discovered that water is a compound of hydrogen and oxygen, that sulfur is an element and that diamond is a form of carbon.

In 1774, Joseph Louis Proust discovered the law of multiple proportions, by which, a chemically pure substance always contains the same set of elements combined together in a definite proportion by weight. He also verified that water always has a fixed ratio of hydrogen to oxygen, regardless of its source.

John Dalton extended Proust’s work and converted the ancient Greek atomic philosophy into a scientific theory. His book, A New System of Chemical Philosophy [63,64], was the first application of atomic theory to chemistry. Dalton proposed that atoms are not infinite in variety; each element possesses a unique kind of atom. Proposing that all the atoms of a given element have the same fixed mass, he concluded that elements react in definite proportions to form compounds because their constituent atoms react in definite proportion to produce compounds. He then tried to figure out the masses for well-known compounds.

In 1809, in his memoir [65,66], Joseph-Louis Gay-Lussac discovered that at a constant temperature and pressure, gasses always combine in simple numerical proportions by volume. He wrote, *Thus it appears evident to me that gases always combine in the simplest proportions when they act on one another; and we have seen in reality in all the preceding examples that the ratio of combination is 1 to 1, 1 to 2 or 1 to 3…*

Gay-Lussac’s work raised the question of whether atoms differ from molecules and, if so, how many atoms and molecules are in a volume of gas.

Avogadro, building on Dalton’s efforts, solved the puzzle, but his work was ignored for 50 years. He proposed that the atoms of elementary gasses form molecules rather than existing as separate atoms, as Dalton believed, and that equal volumes of gasses contain equal numbers of molecules under the same conditions. This hypothesis proved useful in determining atomic and molecular weights, led to the concept of the mole and explained why only half the volume of oxygen is necessary to combine with a volume of carbon monoxide to form carbon dioxide. Each oxygen molecule has two atoms, and each atom of oxygen joins one molecule of carbon monoxide as follows: $2CO+O_{2}=2CO_{2}$.

The mole was initially defined as a weight in grams equal to the molecular weight of the substance in the atomic unit. It was used for quantitatively describing the composition of substances and performing calculations involving the mass and number of particles. In 1991, the mole was redefined as the amount of substance containing exactly N_A_ elementary entities, where N_A_ = 6.02214076 × 10^23^ is the Avogadro number.

**Figure 21:**
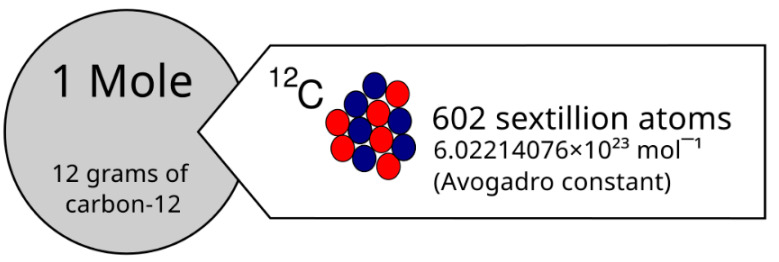
One mole of carbon C-12.

The mole concept is crucial for quantitatively describing the composition of substances and performing calculations involving the mass and number of particles. (See Figure 21).

A balanced chemical equation represents a chemical reaction. Elements are represented using their element symbols and the same number and type of atoms are present on both sides of the reaction. For example:

(7) $$4\;FeS+7\;O_{2}\to 2\;Fe_{2\;}O_{3}\;+4\;SO_{2}\;$$

(8) $$3\;CaCl_{2}\;+2\;Na_{3}\;PO_{4}\;\to Ca_{3}\;{\left(PO_{4}\;\right)}_{2}\;+6\;NaCl$$

(9) $$6\;CO_{2}\;+6\;H_{2}\;O\to C_{6}\;H_{12}\;O_{6}\;+6\;O_{2}\;$$

where (7) describes the iron sulfide combustion, (8) the calcium phosphate precipitation and (9) the photosynthesis.

Equations (7)–(9) illustrate the important concept of valence introduced in 1868. It determines the number of other atoms with which an atom of an element can combine. The valence of hydrogen and sodium is 1, the valence of calcium is 2, of iron is 3, of carbon is 4 and of phosphorus is 5. Later, the theory of valence was reformulated in terms of electronic structures. In various compounds, the atoms can exchange or share electrons in order to form stable valence shells with two or eight electrons. Therefore, the elements in different compounds may have a variable positive or negative valence. For example, in reaction (7), sulfur exhibits the valence 4 and −3.

**Figure 22:**
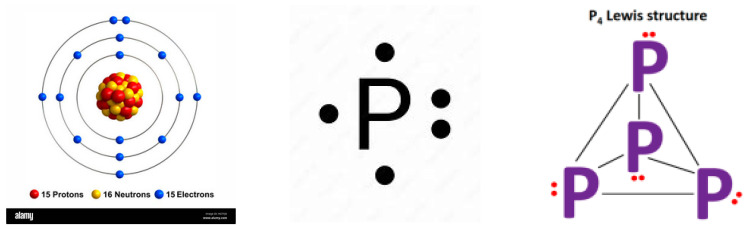
Phosphorus electronic stricture, Lewis’ diagram and a tetrahedral P_4_ molecule.

Phosphorus, which has an atomic number of 15, has fifteen electrons, two in the first energy level (1s^2^), eight in the second energy level (2s^2^ and 2p^6^), and five in the third energy level (3s^2^ and 3p^3^). Phosphorus is very reactive and can have different valence in different compounds. It can use single bonds (sharing a pair of valence electrons) or double bonds (sharing four valence electrons). Such bonds are represented by lines on the Lewis’ diagrams [66,67], and dots represent the valence electrons not used to create a bond. In nature, one finds white phosphorus, whose chemical symbol is P_4_ (See Figure 22).

The important argument in favor of the atomistic theory of nature was given by Dimitri Mendeleev [66,68]. He organized elements in a table based on atomic weight and similar chemical properties such as valence, etc. He left gaps in places where he believed unknown elements would eventually find their place. Remarkably, he even predicted the likely properties of three of these potential elements. The subsequent confirmation of many of his predictions during his lifetime brought him fame as the founder of the periodic law.

**Figure 23:**
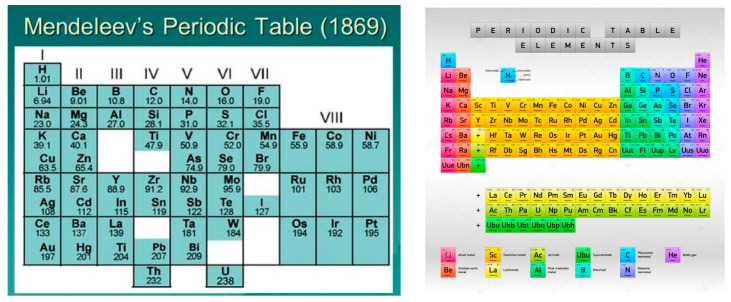
Periodic tables in 1869 and the modern table in which atomic number instead of mass is used.

His work laid the foundation for our modern understanding of the periodic table, which now orders elements by increasing atomic number. Mendeleev’s groundbreaking work significantly advanced the field of chemistry (See Figure 23).

In chemistry and the kinetic theory of gasses, atoms and ions were used as indivisible units. In 1865, Joseph Loschmidt [66], using various available rough experimental data, estimated that the diameter of an atom was approximately 10^−8^ cm. His estimation of the Avogadro constant was also close to the present accepted value.

## 8. From Faraday to Quantum Mechanics

Scientists ignored the nature of the forces binding atoms together in a molecule. Faraday [69] discovered that electrical forces existed inside the molecule. He had produced an electric current and a chemical reaction in a solution with the electrodes of a voltaic cell. No matter what solution or electrode material he used, a fixed quantity of current sent through an electrolyte always caused a specific amount of material to form on an electrode of the electrolytic cell. Faraday concluded that each ion of a given chemical compound has exactly the same charge and that ionic charges are integral multiples of a single unit of charge, never fractions. The unit of charge that releases one gram-equivalent weight of a simple ion is called a *faraday* (F) in his honor. For example, one faraday of charge passing through water releases one gram of hydrogen and eight grams of oxygen.

**Figure 24:**
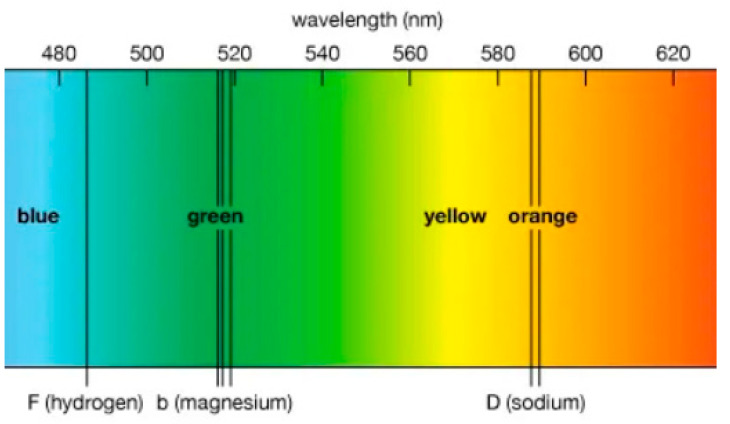
The visible solar spectrum, ranging from the shortest visible wavelengths (violet light, at 400 nm) to the longest (red light, at 700 nm). Shown in the diagram are prominent Fraunhofer lines, representing wavelengths at which light is absorbed by elements present in the atmosphere of the Sun.

By far the richest clues about the structure of the atom came from spectral line series. Isaac Newton already allowed sunlight to pass through a small, circular hole and fall on a prism, which produced a rainbow of colors that he called a spectrum. He explained that light consists of different rays, some more refrangible than others. Joseph von Fraunhofer made a significant leap forward in the early 1800s. Mounting a particularly fine diffraction grating on a telescope, he had discovered hundreds of dark lines in the spectrum of the Sun. He labeled the most prominent of these lines with the letters A through G. They are now called Frauenhofer lines. Stars emit light from their photospheres. When this light passes through the outer atmosphere (chromosphere), certain atoms absorb specific wave lengths. These absorbed wavelengths correspond to the energy levels of electrons in the atoms, which gives information about the composition of the star [66] (See Figure 24).

Around 1860, Gustav Kirchhoff heated different elements to incandescence in order to study the differently colored vapors. Observing these vapors through a spectroscope, he discovered that each element has a unique and characteristic pattern of spectral lines. Each element produces the same set of identifying lines, even when it is chemically combined with other elements [66].

In 1865, Maxwell [70] unified the laws of electricity and magnetism and concluded that light is an electromagnetic wave. Maxwell’s theory failed to describe spectral lines and the fact that atoms do not lose all their energy when they radiate light.

**Figure 25:**
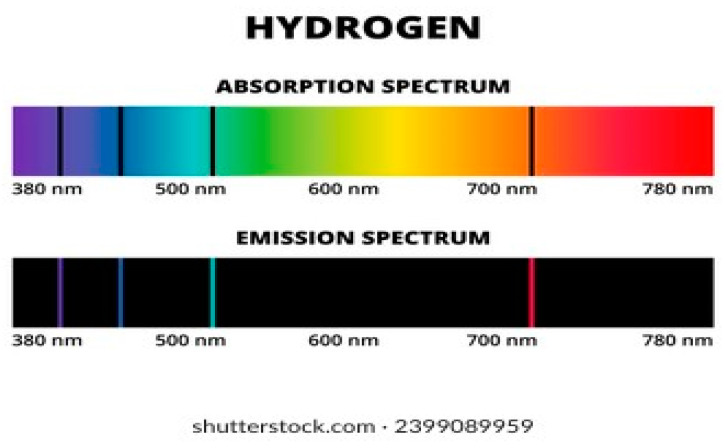
Balmer series of hydrogen visible spectral lines.

In 1853, Anders Ångström measured the four visible spectral lines of hydrogen to have wavelengths 656.21, 486.07, 434.01 and 410.12 nm (See Figure 25).

**Figure 26:**
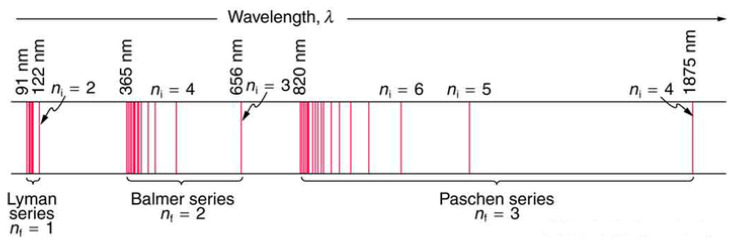
Full hydrogen spectrum including infrared and ultraviolet.

In 1985, Johann Balmer, a Swiss secondary-school mathematics teacher found a constant relation between the wavelengths of the element’s four visible lines [71], as follows:

(10) $$\lambda_{m}=b(\frac{m^{2}}{m^{2}-4})$$

where b = 364.56 nm and m = 3, 4, 5, 6. He predicted that other lines existed in the ultraviolet spectrum that corresponded to m ≥ 7 and some of them had been discovered. The Balmer formula is a special case of a more general formula discovered by Johannes Rydberg in 1890, which follows:

(11) $$\frac{1}{\lambda }=R_{H}(\frac{1}{{n_{1}}^{2}}-\frac{1}{{n_{2}}^{2}})$$

where R_H_ = 1.09737 m^−1^ is the Rydberg constant and *n*_2_ > *n*_1_ are integers. The value of *n*_1_ defines a particular series of spectral lines. For Lyman series *n*_1_ = 1, for Balmer series *n*_1_ = 2, for Paschen series *n*_1_ = 3 etc (See Figure 26).

In 1897, J. J. Thomson discovered that the electron was a carrier of electricity in cathodic rays and found that the mass of the electron was very small, merely 1/1836 that of a hydrogen ion, and the scientists realized how electric current could flow through copper wires. In deriving the mass-to-charge ratio, Thomson calculated the electron’s velocity. It was 1/10 the speed of light, thus amounting to roughly 30,000 km (18,000 miles) per second. The electron was the first subatomic particle identified, the smallest and the fastest bit of matter known at the time. In 1909, American physicist Robert Andrews Millikan directly measured the charge of the electron to be 1.602 × 10^−19^ coulomb [66].

Wilhelm Conrad Röntgen had discovered X-rays in 1895. Like Thomson’s discovery of the electron, the discovery of radioactivity in Uranium by French physicist Henri Becquerel in 1896 forced scientists to radically change their ideas about atomic structure. Radioactivity demonstrated that the atom was neither indivisible nor immutable. In 1898, Pierre and Marie Curie discovered the strongly radioactive elements polonium and radium, which occur naturally in uranium minerals. In 1899, Ernest Rutherford showed that radioactive substances emit more than one kind of radiation. Beta rays are beams of electrons and alpha rays are beams of positively charged helium ions. A third kind of radiation was identified and called the gamma rays; it was not deflected by magnets and was much more penetrating than alpha particles. Gamma rays were later shown to be a form of electromagnetic radiation, similar to light or X-rays, but with much shorter wavelengths [66].

In 1902, Rutherford and English chemist Frederick Soddy discovered that radioactivity was associated with changes inside the atom that transformed thorium into a different element. They found that thorium continually generates a chemically different substance that is intensely radioactive and gradually disappears. Watching the process, they discovered exponential radioactive decay, which states that a fixed fraction of the element will decay in each unit of time. For example, half of the thorium product decays in four days, half the remaining sample in the next four days, and so on.

In his gold foil experiments, Rutherford observed that only very few of the alpha particles in his beam were scattered by large angles after striking the gold foil, while most passed completely through. He concluded that the gold atom’s mass must be concentrated in a tiny dense nucleus and proposed a model of the atom as a miniature solar system, with electrons orbiting around a massive nucleus consisting only of protons and occupying only a very small part of the atom. However, according to classical electrodynamics, the model was unstable because the electron would gradually lose energy and spiral into the nucleus. No electron could thus remain in any particular orbit indefinitely. The model also disagreed with the Mendeleev table because the neutron was not discovered yet [66].

In 1905, Einstein postulated that the exchanges of energy between light and matter are quantized. In other words, a monochromatic light with frequency ν behaves like a beam of photons carrying energy E = hν and a linear momentum p = hk (k = 1/λ), and thus, the energy of an electron in an atom can change only in multiples of hν, where h is a Planck constant, h = 6.6 × 10^−34^ (joule ∙ second). Planck introduced this constant in 1900, in a formula explaining light radiation emitted from heated bodies. He postulated that energy can only be emitted or absorbed in discrete amounts of hν, which he called quanta.

In 1913, Henry Moseley found that each element radiates X-rays of a different and characteristic wavelength. The wavelength and frequency vary in a regular pattern according to the charge on the nucleus. He called this charge the atomic number. His results, Balmer and Rydberg’s spectral series and Planck’s and Einstein’s quantized exchanges of energy between light and matter inspired Bohr to postulate the first successful model of the hydrogen atom.

In 1913, Niels Bohr modified the Rutherford model by requiring that electrons move in orbits of fixed size and energy. The energy of an electron depends on the size of the orbit and is lower for smaller orbits. Radiation can occur only when the electron jumps from one orbit to another. The atom will be completely stable in the state with the smallest orbit, since there is no orbit of lower energy into which the electron can jump.

**Figure 27:**
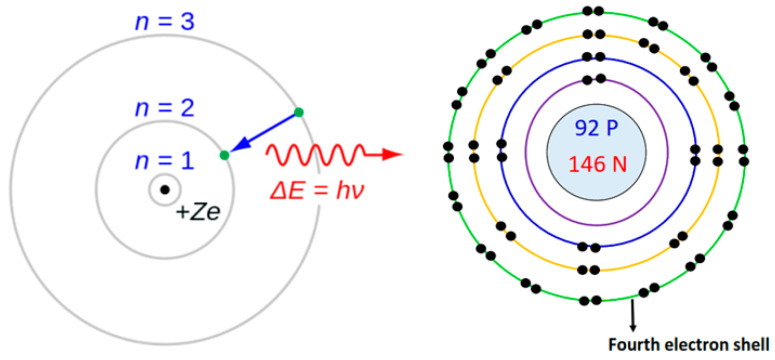
Bohr model of an atom. Maximum number of electrons: 2 in the first shell, 8 in the second shell and 18 in the third shell.

Bohr assumed that the angular momentum of the electron is quantized, i.e., it can have only discrete values and electrons obey the laws of classical mechanics by traveling around the nucleus in circular orbits. Because of the quantization, the electron orbits have fixed sizes and energies. The energy of an electron in the *n*-th shell is given by E(*n*) = −13.6/*n*^2^ eV. The energy of the emitted photon hν = ΔE = E(*n*_2_) – E(*n*_1_) agrees completely with the Balmer–Rydberg Formula (11) and Bohr was able to calculate the value of the Rydberg constant [72]. Bohr’s model does not work for systems with more than one electron (See Figure 27).

At the same time, J. J. Thomson found that a beam of neon atoms subjected to electric and magnetic forces split into two parabolas instead of one on a photographic plate. Chemists had assumed the atomic weight of neon was 20.2, but the traces on Thomson’s photographic plate suggested atomic weights of 20.0 and 22.0, with the former parabola much stronger than the latter. He concluded that neon consisted of two stable isotopes, primarily neon-20, with a small percentage of neon-22. Eventually a third isotope, neon-21, was discovered in very small quantities. He disproved Dalton’s assumptions that all atoms of an element have an identical mass and that the atomic weight of an element is its mass. Today the atomic weight of an element is recognized as the weighted average of the masses of its isotopes.

As we explained above, the light, which was initially thought to be a wave, was found to have particle-like properties. In 1924, Louis de Broglie proposed the wave nature of electrons and suggested that all matter has wave properties. De Broglie wavelength follows *λ*_B_ = h/p, where p is a particle momentum and h is a Planck constant. For example, a beam of electrons can be diffracted just like a beam of light or a water wave. The wave-like behavior of matter has been experimentally demonstrated, first for electrons in 1927, and later for neutrons, neutral atoms and molecules in numerous experiments. This concept is known as the wave–particle duality and inspired Erwin Schrödinger in his formulation of wave mechanics, which evolved into the modern quantum mechanics. Wave–particle duality is sometimes incorrectly interpreted as a particle that is **at the same time** wave and particle and that the electron can be here and a meter away at the same time.

Bohr’s atom and wave mechanics were the last attempts to explain atomic and subatomic physics using semi-classical models. Classical mechanics was created as an abstraction from our everyday observations. The objects had attributive properties which could be measured with increasing (theoretically unlimited) precision. Similarly, during their motion in the absolute Newtonian space, at each moment of absolute time, they had precise positions, energies, linear and angular momenta in a chosen inertial reference frame. Of course, the measurement of the distance was only direct when a measuring stick, rod or tape could be used; other distances could only be determined using Euclidean geometry and triangulation. Nevertheless, measurements by definition were noninvasive; this means they did not change the value of the physical observable they wanted to measure.

According to the law of universal gravitational attraction, distant masses should influence each other’s motions instantaneously across empty space, which was contrary to everyday experience and Aristotelean physics. Leibniz and Huygens called it the unacceptable interaction at the distance. Newton insisted that his model is an abstract mathematical model consistent with the observations and that it is sufficient. With the discovery of electromagnetism and the contributions of Faraday and Maxwell, it became clear that space is not empty and that the electromagnetic waves carry energy and linear momentum and can mediate the interaction between distant bodies. As Planck and Einstein demonstrated, the exchange of energy between the wave and matter were quantized. In order to explain this, modern quantum mechanics was created.

## 9. From Quantum Mechanics to the Standard Model

Quantum mechanics is an abstract mathematical theory allowing the deduction of probabilistic predictions about observed phenomena and outcomes of various experiments. There are different interpretations of quantum mechanics. For me, the most consistent is the statistical contextual interpretation [73,74,75,76,77,78,79]. An ensemble of identically prepared physical systems is described by a state vector (wave function) $|\psi \rangle$ in a Hilbert space H. A measured physical observable A is represented by a self-adjoint operator $\hat{A}$ acting in H, whose eigenvalues $\lambda_{i}$ are the only possible outcomes of the measurement and the expectation value $E(A)=\sum_{i}\lambda_{i}p(\lambda_{i})=\langle \psi |\hat{A}|\psi \rangle$. In contrast to classical mechanics, there exist incompatible physical observables which cannot be measured with arbitrary precision at the same time and are represented by non-commuting operators, e.g., for the position and the corresponding linear momentum component, we have $[\hat{x},{\hat{p}}_{x}]=i\hbar$, where ℏ = *h*/2π.

Measurement outcomes in quantum mechanics are not preexisting values of physical observables recorded with errors by measuring instruments. Measurement outcomes are created in the interaction of a measuring instrument with physical systems. Since the speed of light is a universal constant, coordinates of an event in special relativity are determined using a radar method. For example, in one spatial dimension, we obtain the following:

(12) $$x=c(t_{2}-t_{1})/2;\;t=(t_{2}+t_{1})/2$$

where *t*_1_ and *t*_2_ are the respective times of sending and receiving a reflected light signal. Physical objects are not material points; thus, neither in classical physics nor in quantum physics exist perfectly precise position measurements. In the macroscopic physics the radar method can be considered non-invasive, in spite of the fact that light is an electromagnetic wave carrying energy and momentum. In the quantum domain, we do not see atoms, electrons and “photons” but only spots on photographic plates, traces in cloud chambers or clicks on detectors. Therefore, the radar method cannot be used. In addition, for precise localization we have to use light signals with shorter wavelengths and a higher energy of “photons”, and we could destroy the quantum system being measured. In fact, in collisions of gamma rays with electrons, several particle–antiparticle pairs can be produced. In order to describe the processes in which particles may be created and annihilated, quantum electrodynamics (QED) and quantum field theory (QFT) were created. In these sophisticated mathematical theories, only the linear momentum, spin and some additional quantum numbers are valid observables. In quantum mechanics, one defines an operator representing the position measurement; in QED and in QFT, there is no such operator.

As we can read in the article in the Stanford Philosophical Encyclopedia [80], quantum Field Theory (QFT) is the mathematical and conceptual framework for contemporary elementary particle physics. It is also a framework used in other areas of theoretical physics, such as condensed matter physics and statistical mechanics. In a rather informal sense, QFT is the extension of quantum mechanics (QM), dealing from particles to fields, i.e., systems with an infinite number of degrees of freedom.

QFT is a complicated mathematical model [80,81]. Its equations cannot be solved, and to explain experimental data, one constructs various semi-empirical models inspired by QFT. We explain below, in a simplified way, how QFT and the Standard Model are used to make quantitative predictions in particle physics.

A quantum field is an operator-valued distribution defined at each point of the four-dimensional Minkowski space-time. Each free quantum field is associated with a specific particle (excitation). The states of the quantum field are *n*-particle states (*n* changing from 1 to infinity). If one has k-interacting different quantum fields, they can only describe how the collision of two particles changes their linear momenta and energies and which other particles described by these k-fields can be created as the effect of the interaction. In general, at a given initial total energy, several possible final states may be created and observed. The probability of observing a particular final state $|f\rangle$ from the initial $|i\rangle$ is given by $P_{if}=|\langle f|\hat{S}|i\rangle {|}^{2}$, where $\hat{S}$ a unitary operator, being a complicated nonlinear function of interacting fields and their partial derivatives. If $\hat{S}$ depends on a small parameter *g*, called a coupling constant, one replaces $\hat{S}$ (g) by an infinite series in powers of g, with coefficients which are complicated analytical expressions and products of creation and annihilation operators. Finally, one uses only one or two non-trivial first terms of this series to calculate an approximated value of $P_{if}(g,..)\approx |\sum_{m}f_{m}(g,..){|}^{2}$, where $f_{m}(g,\ldots )$ are complex valued functions of the coupling constant and quantum numbers, describing the corresponding initial and final states. These functions are graphically represented by Feynman graphs and are often incorrectly interpreted as the images of the physical process happening during the interaction [80,81,82,83].

**Figure 28:**
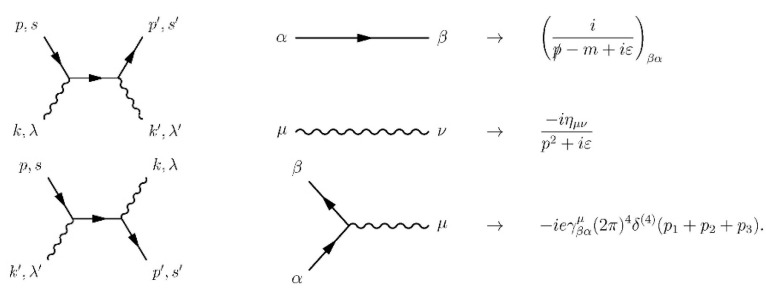
Feynman graphs as mnemonic tools to account for the important mathematical terms to be included in the calculations in QED.

In QED, we have a fermionic field corresponding to electrons and positrons, and bosonic field corresponding to γ particles (See Figure 28).

Several integrals in the perturbative expansion of the transition probabilities discussed above are divergent and specific renormalization and regularization procedures [83] are necessary to extract meaningful quantitative predictions to be compared with experimental data. Considering all that, it is surprising how well these predictions agree with the data. The infinities arrive because the fields are defined in a continuous space-time and we are dealing with point-like charges and masses. It would be much more elegant to construct a theory which does not require any renormalization. This was the opinion of Dirac, who at the end of his book wrote, “the difficulties being of a profound character can be removed only by some drastic change in the foundations of the theory, probably a change as drastic as the passage from Bohr’s orbit theory to the present quantum mechanics” [84]. Feynman was also dissatisfied with the renormalization/regularization procedures [82].

The neutron was only discovered by John Chadwick in 1932. When Beryllium was bombarded with α particles (helium ions), neutrons were created, 9Be + 4 α → 12C + *n*. Also, in 1932, the positron (an anti-electron predicted by Dirac) was discovered by Carl David Anderson in the experiments with cosmic rays in a Wilson cloud chamber. Charged particles moving across cloud chambers are leaving visible traces. The Lorentz force **F,** acting on a charged particle, is given by the following equation: **F** = q (**E** + **v** × **B**), where q is the charge of the particle in (C), **E** is the electric field vector in (V/m), **v** is the velocity vector of the particle in (m/s) and **B** is the magnetic field vector in tesla (T). By applying external magnetic and electric fields on a charged particle moving across the cloud chamber, one may determine its mass and charge.

Cosmic rays are high-energy particles that move through space at nearly the speed of light. They originate from various sources, including the Sun, supernova explosions, distant galaxies, etc. When cosmic rays hit the Earth’s atmosphere, they produce showers of secondary particles, some of which reach the surface. In 1932, one could think that all building ingredients of matter were discovered. This was not true. The discovery of muon in 1937 was followed by the discovery of pions, kaons, many other particles and resonances in cosmic rays or in high-energy scattering experiments, made possible due to the construction of different particle accelerators and colliders.

More and more precise particle detectors were developed, including bubble chambers, wire chambers, spark chambers, wire proportional chambers, drift chambers, silicon detectors and various calorimeters. Calorimeters measure the energy of particles. Particles enter the calorimeter and initiate a particle shower in which their energy is deposited and measured. It is the most practical way to detect and neutral particles produced in an interaction. Calorimeters also allow to calculate “missing energy”, which can be attributed to particles that rarely interact with matter and escape the detector, such as neutrinos.

In the 1950s, in the interactions of pions and neutrons in the atmosphere, “strange particles” were discovered, including kaon (K), lambda (Λ) and sigma (Σ), which exhibited unusual properties in their production and decay. Another peculiar feature was that they were always produced in pairs. To explain this, a new conserved quantum number, strangeness, was introduced. Strange particles are produced by strong interactions at a high rate, but they decay slowly, only via weak interactions [85]. Their half-lives are in the range 10^−10^ s to 10^−8^ s, and they can be studied using bubble chamber photographs.

**Figure 29:**
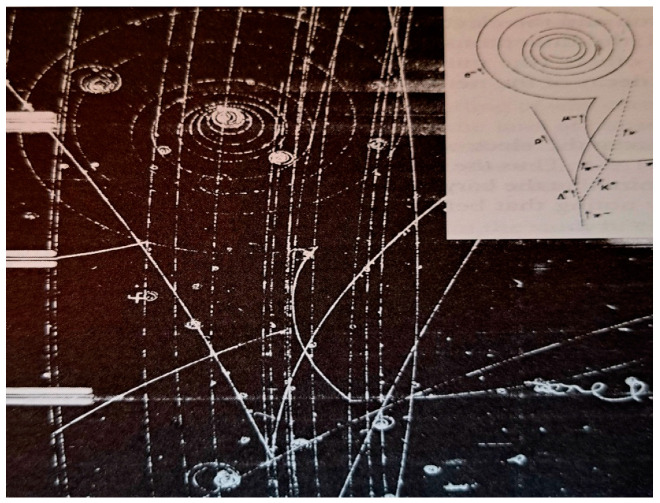
The bubble chamber photography shows many events after a high-energy collision of $\pi^{-}$ with a proton (12); the insert is a drawing of identified tracks [85].

For the example on the photograph below (See Figure 29), from the bubble chamber, we can see the production of K^0^ and Λ^0^ particles followed by their successive decays into charged particles leaving the visible traces, as follows:

(13) $$\pi^{-}+p\to K^{0}+\Lambda^{0}\Rightarrow \;\Lambda^{0}\to \pi^{-}+p\;and\;K^{0}\to \pi^{+}+\mu^{-}+{\bar{\nu }}_{\mu }$$

Elementary particles and resonances have a wide range of lifetimes, depending on their specific properties. The lifetimes range from that of the neutron 10^−3^ s to 10^−23^ s. If the lifetime of a particle is of the order of 10^−23^, then traveling at the speed of light, this particle could only travel about 10^−15^ m, or about the diameter of a proton, before decaying.

Therefore, such lifetimes are typically determined using the energy-time uncertainty principle as follows:

(14) $$\Delta E\Delta t\geq \frac{\hbar }{2}$$

which suggests that for particles with extremely short lifetimes, there will be significant uncertainty in the measured energy. By measuring the total invariant mass of the decay products of an unstable particle, one obtains a Breit–Wigner distribution [86]. The width of this distribution at half-maximum is labeled Γ = 2ΔE. For example, in the collisions of electrons with protons:

(15) $$e^{-}+p\to e^{-}+\Delta^{+}\Rightarrow e^{-}+\pi^{+}+n$$

we detect only electrons and $\pi^{+}+n$. We discover that they are decay products of $\Delta^{+}$ by studying the distribution of the invariant total mass Z, as follows:

(16) $$Z={\left({\left(E_{\pi }+E_{n}\right)}^{2}+{\left({\vec{p}}_{\pi }+{\vec{p}}_{n}\right)}^{2}c^{2}\right)}^{1/2}$$

**Figure 30:**
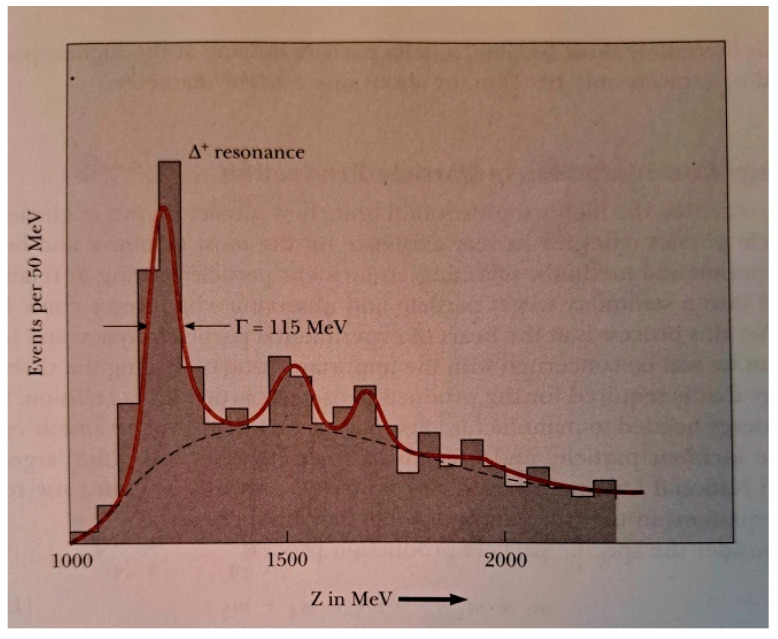
Histogram of invariant mass proving the existence of elementary particle $\Delta^{+}$ [85].

On the Figure 30 below, we can see the histogram of values of Z for all observed collision events, allowing us to estimate the mass and the half-life time of the unstable particle $\Delta^{+}$.

The broad background (dashed curve) is produced by direct events in which no $\Delta^{+}$ was created. The sharp peak Z = 1232 MeV corresponds to the events in which $\Delta^{+}$ was formed and decayed. Its lifetime is extremely short, $\Delta t\approx \frac{\hbar }{2E}=\frac{\hbar }{\Gamma }=5.7\times 10^{-24}s$ [85].

Hundreds of new particles and resonances were identified using this method. Following Pythagoreans, Aristotle, Democritus and the Mendeleev physicists succeeded in reducing the number of “elementary building blocks of matter” to a relatively small number in the Standard Model, which we are going to review shortly below [87,88,89,90,91].

**Figure 31:**
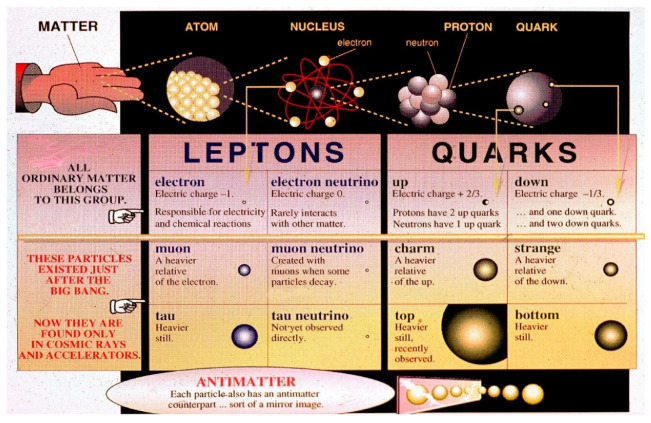
Building blocks of matter according to the Standard Model.

Pythagoreans believed that natural numbers played an important role in nature. By chance or not, they also play an important role in the Standard Model (SM) (See Figure 31). In SM we have:

- Four fundamental forces, strong, weak, electromagnetic, gravitation.
- Six leptons, six quarks in three colors, four gauge bosons; one Higgs (God’s particle).
- White baryons (three quarks), p—uud, *n*—udd…; mesons (quark-antiquark).
- Symmetry groups, SU(3), SU(6)…; triplets, octets, decuplets…

Fermions are fundamental particles with no measurable internal structure. They include quarks (which make up protons and neutrons) and leptons (such as electrons and neutrinos). Fermions have half-integer spins. Quarks are the building blocks of hadrons (protons, neutrons and mesons). They interact via strong forces and come in six flavors, up, down, charm, strange, top, and bottom. Bosons mediate forces. The Higgs boson (discovered in 2012) gives mass to other particles. Baryons consist of three quarks, while mesons have one quark and one antiquark.

Similarly to Mendeleev, who regrouped elements according to their properties, the physicists regrouped the discovered elementary particles into specific “families” and “multiplets”. Particles are sorted into groups as mesons or baryons. Within each group, they are further separated by their spin angular momentum.

**Figure 32:**
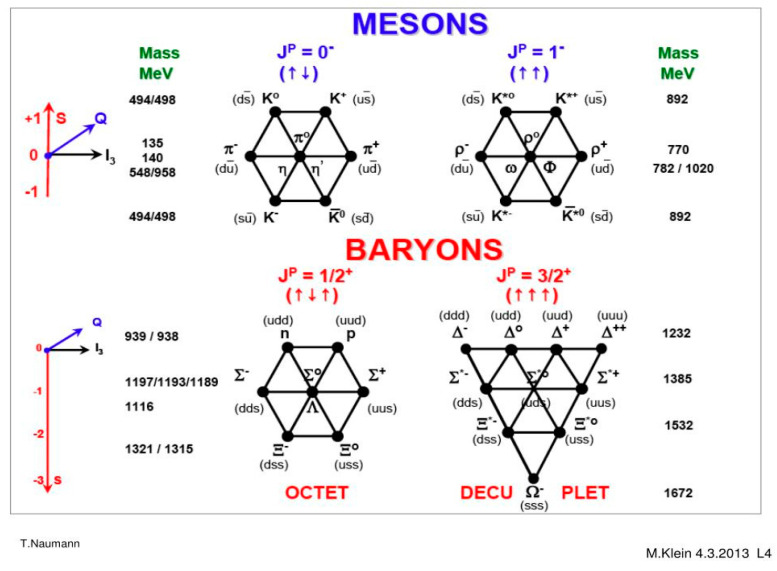
Meson nonets, baryon octet and decuplet.

Symmetrical patterns appear when groups of particles have their strangeness plotted against their electric charge. This is the most common way to make these plots today, but originally, physicists used an equivalent pair of properties called hypercharge and isotopic spin, the latter of which is now known as isospin. The symmetry in these patterns is a hint of the underlying symmetry of the strong interaction between the particles themselves. This led to the discovery of SU(3) and SU(6) symmetries and to the successive quark models [88,89,90] (See Figure 32). 

In the plots above, points representing particles that lie along the same horizontal line share the same strangeness, s, while those on the same left-leaning diagonals share the same electric charge, q (given as multiples of the elementary charge). Pythagoreans would be happy to see their sacred number **10** represented by Tetractys in baryon and anti-baryon, spin 3/2, decuplets and the **four** fundamental forces of Nature.

We are talking about the “building blocks of matter” and draw nice diagrams, but in fact, we are not allowed to make any mental pictures. The SM is a complicated abstract and semi-empirical mathematical model containing 26 free parameters. It contains algorithm “recipes”; how to make calculations and how to compare them with the data gathered by different counters and detectors. Nevertheless, the SM allows us to explain several regularities in these experimental data and to make verifiable predictions confirmed by subsequent experiments.

Free stable quarks do not exist in nature. By 1977, physicists had isolated five of the six quarks in the lab (up, down, strange, charm and bottom), but it was not until 1995 that researchers at the Fermilab National Accelerator Laboratory in Illinois “found” the top quark. Searching for it had been as intense as the later hunt for the Higgs boson. The top quark was so hard to produce because it is about eighty thousand times heavier than up quarks, meaning it required a lot more energy to make using particle accelerators.

We explain below in some detail how the hadron–hadron strong collision is described in the Standard Model. Quantum Chromodynamics (QC) [90,91] is a theory of strong interactions between quarks and gluons, which is a generalization of QED. If $\left|i\right\rangle$ is an initial state vector of *n* free quarks, a probability of finding a final state $\left|f\right\rangle$ of *m* free quarks is defined as $P_{if}=|\langle f|S|i\rangle {|}^{2}$. The S matrix is replaced by a perturbative series and only a few first terms of this series are evaluated and used as an approximation of **P**_if_, as follows:

(17) $$P_{\mathrm{if}}(s,t,\mathrm{quantum}\;\mathrm{numbers},\ldots )\approx |\sum \left(\int products\;\;of\;Feynmann\;graphs\right){|}^{2}$$

**Figure 33:**
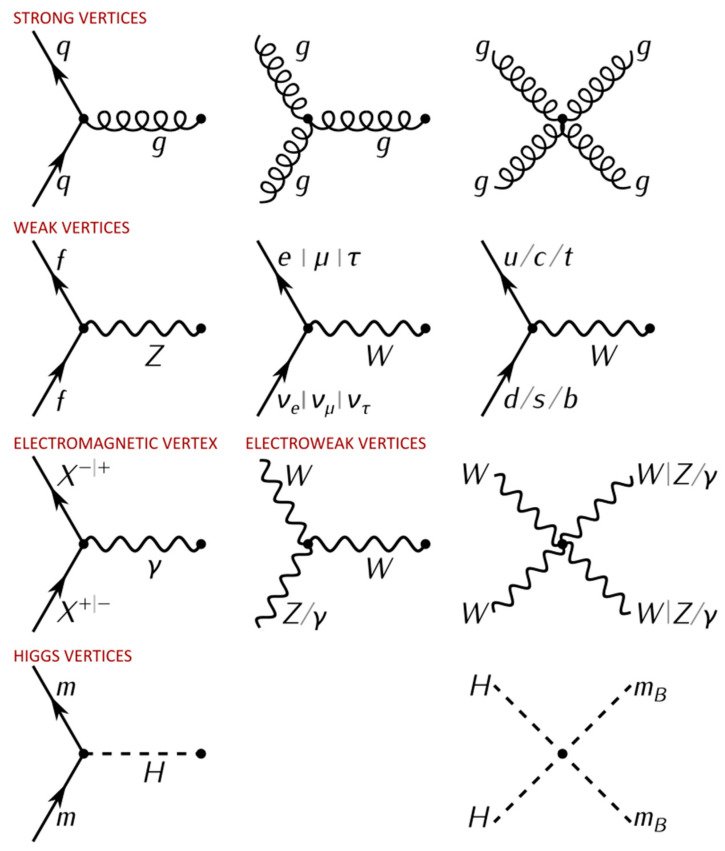
Interactions in the Standard Model. All Feynman diagrams in the model are built from combinations of these vertices; q is any quark, g is a gluon, X is any charged particle, γ is a photon, f is any fermion, m_B_ is any boson with mass. In diagrams with multiple particle labels separated by /, one particle label is chosen. In diagrams with particle labels separated by |, the labels must be chosen in the same order. For example, in the four boson electroweak case, the valid diagrams are WWWW, WWZZ, WWγγ, WWZγ. The conjugate of each listed vertex (reversing the direction of arrows) is also allowed [90].

All Feynman graphs are built using the following elementary vertices displayed below [90] (See Figure 33). 

Colliding hadrons are represented by free quark states via universal semi-empirical *parton distribution functions* (PDFs) [92]. PDFs describe the probability distributions of quarks and gluons (collectively called partons) inside a hadron. They provide information about the momentum fraction carried by each parton at a given energy scale. PDFs are universal, meaning they are process-independent and apply to all high-energy interactions involving hadrons. PDFs are used in collider experiments (e.g., LHC) to predict cross sections for various processes. Uncertainties in PDFs directly affect the predicted cross sections. PDFs have associated uncertainties due to experimental data limitations and theoretical assumptions. These uncertainties are quantified using error bands. Collider observables (e.g., Higgs boson production) depend on PDFs.

Then, using (17), various probabilities are calculated. Hadronization, how at the end, free quarks recombine to form final particles and resonances, cannot be described rigorously in the SM. No exact theory for hadronization is known, but two empirical models for parameterization are used within event generators which simulate particle physics events [93].

The SM falls short of being a complete theory; it does not explain baryon asymmetry, gravity (as described by general relativity), or dark energy. It lacks a viable dark matter particle and does not account for neutrino oscillations and their masses. Moreover, estimates of the values of quark masses depend on the version of QCD used to describe quark interactions. Quarks are always confined in an envelope of gluons that confer a vastly greater mass to the mesons and baryons, so values for quark masses cannot be measured directly. Since their masses are so small compared to the effective mass of the surrounding gluons, slight differences in the calculation lead to large differences in the masses.

In LHC experiments, millions of collision events are produced, and completely different methods have to be used in order to extract meaningful information about the created particles, quarks and their lifetime. These methods are based on the interplay of the semi-empirical theoretical models, sophisticated computer data processing and simulations. Experiments use trigger systems to select interesting events for further analysis. Only a fraction of the data is stored, reducing the volume significantly. Experiments rely on powerful computing clusters to process and analyze data. Algorithms compress data without losing essential information. Lossless compression techniques are used.

**Figure 34:**
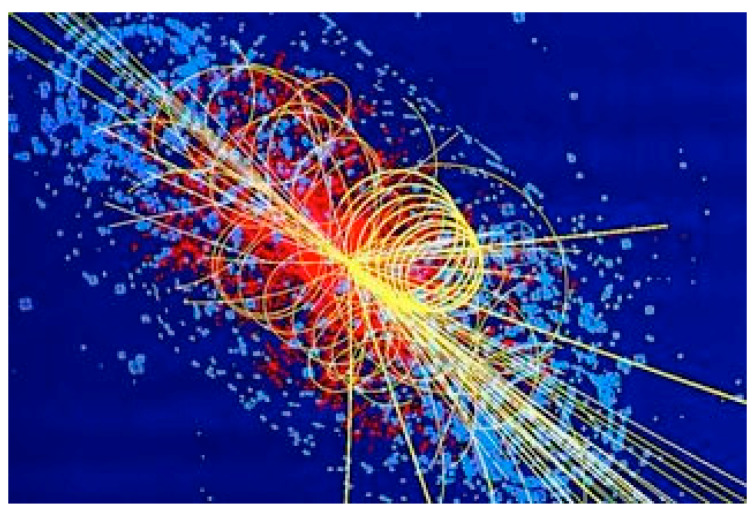
Simulation showing the production of the Higgs boson in the collision of two protons at the Large Hadron Collider. The Higgs boson quickly decays into four muons, which are a type of heavy electron that is not absorbed by the detector. The tracks of the muons are shown in yellow. (Image credit: Lucas Taylor/CMS).

Several event generators [94] simulate interesting events, such as the creation of the Higgs boson using the semi-empirical and theoretical inputs and experimental data. Then, particular computer art software creates “event images” for scientists and for the general public (See Figure 34). 

As we can see, the Standard Model and the description of high energy collisions are quite far from the picture of planets playing harmonious music to please the Creator. Therefore, we should be perhaps much humbler.

## 10. Bild Conception of Physical Theory and Modern Neuroscience

As we mentioned in the introduction, Helmholtz, Hertz, Boltzmann and Schrodinger insisted that our models of physical reality, based on our sensorial sensations, are only intellectual constructs of our brain unable to describe nature as it is.

Helmholtz [4,5] had no doubts that laws in nature really existed, but the laws presented in scientific theories were only mental representations of these laws. They were only “parallel” to natural laws, not identical, since our mind does not operate with precise images of real objects but only with the symbols assigned to them [12].

Hertz believed that Helmholtz’s parallelism of laws was impossible if theory were limited to describing observable quantities, because the manifold of the actual universe is greater than the manifold of the universe which is directly revealed to us by our senses.

Only by introducing hidden quantities (concepts that correspond to no perceptions) can Helmholtz’s parallelism of laws become a general principle in physical theory. Such theory should be constrained by causality and simplicity. Namely, if our images are well-adapted to things, the actual relations of things must be represented by simple relations between images… Even a “good model” does not describe reality as it is; it provides just a mathematical symbolic representation involving a variety of elements having no direct relation with observational quantities [6,7,12]. This conception was further developed and promoted by Boltzmann [8] and Schrodinger [9,10].

**Figure 35:**
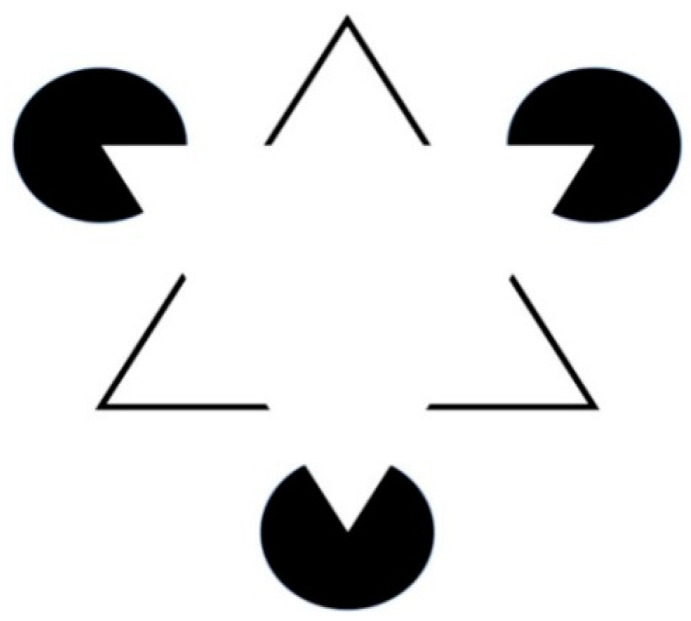
The Kanizsa triangle: the Pac-Man-like shapes give the impression of a triangle in our minds. It seems like a triangle, because we are used to seeing triangles.

**Figure 36:**
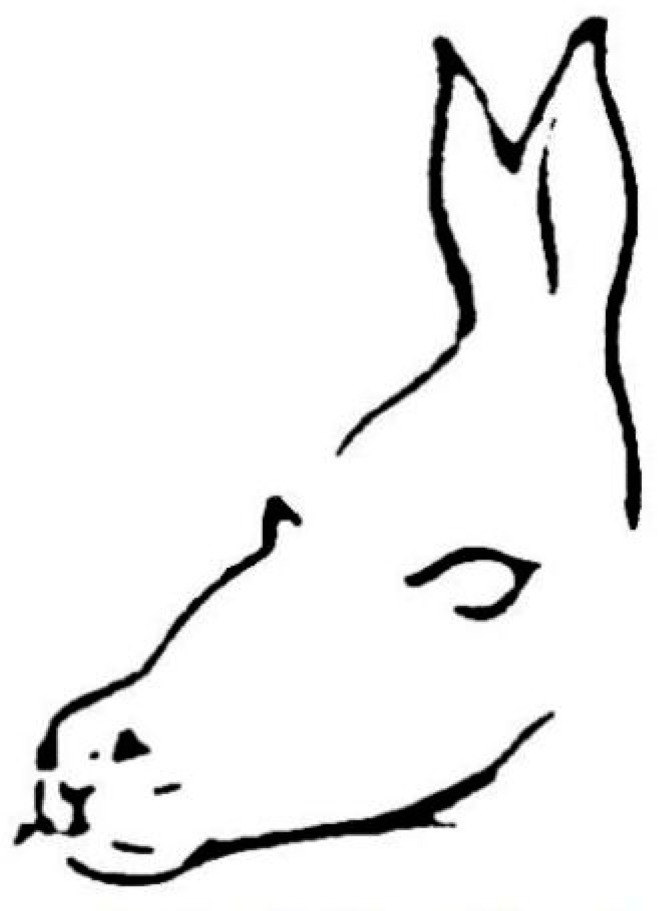
We see a horse’s head or a seal depending on our previous life experiences.

Recent studies in neuroscience [95], which we discuss shortly below, provide additional arguments in favor of the Bild conception, because the physical reality, as we perceive it, is in fact created by our brain. Patrick Cavanagh (GLENDON) argued, “We’re seeing a story that’s being created for us… Most of the time, the story our brains generate matches the real world, but not always”. A detailed explanation and several examples of visual illusions may be found in [95,96,97]. Our brains unconsciously bend our perception of reality to meet our desires or expectations. They fill in gaps using our past experiences creating visual illusions (See Figure 35 and Figure 36). 

**Figure 37:**
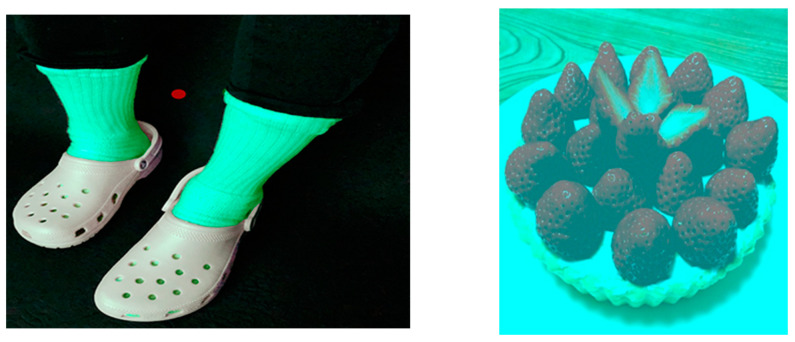
In reality, the Crocs are pink, the pixels in the strawberries are only gray and cyan. *Courtesy of Pascal Wallisch*.

The visual cortex is at the back of our brain; the frontal lobes are the higher-level thinking area dedicated to anticipation and decision-making. Sam Schwarzkopf, a vision scientist at the University of Auckland, says, “we’re not trying to measure wavelengths, we’re trying to tell something about the color and the color is an illusion created by our brain” [95] (See Figure 37). 

Susana Martinez-Conde (SUNY) argues, “We’re not seeing reality. Our vision runs 100 milliseconds behind the real world. Why are we seeing a story… It’s actually an adaptation. We don’t have the necessary machinery to process carefully all the information that we’re constantly bombarded with”.

Adam Hantman, a neuroscientist at Howard Hughes Medical Institute’s Janelia Research Campus, claims, “Our brains like to predict as much as possible, then use our senses to correct, when the predictions go wrong. This is true not only for our perception of motion but also for so much of our conscious experience”. The stories our brain tells us about physical reality are often misleading and are influenced by our life experiences.

Pascal Wallisch, a clinical associate professor at New York University explains, “When an image, event, or some other stimulus is not perfectly clear, we fill in the gaps with our priors, or presumptions. Neuroscience is deeply humbling. We should cultivate a habit of seeking out perspectives, that are not our own”. Political partisans perceive the facts of current events differently, depending on their political beliefs. Their illusions and political thinking do not involve the same brain processes, but they follow the similar overarching way the brain works [95].

**Figure 38:**
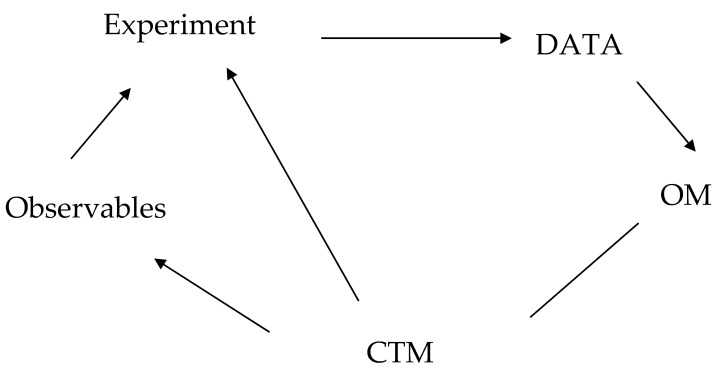
Epistemological cycle, using theoretical model CTM, observables are chosen and an experiment is designed and performed. Regularities in experimental data are discovered and the observational model OM is postulated and tested. An improved CTM is constructed, additional observables are defined and new experiments are designed and performed.

Progress in model building in science follows a self-improving epistemological cycle. We define *physical observables*, design and perform experiments to measure their values. Analyzing experimental data, we discover *empirical laws* and construct an observational *model* (OMs), which are not constrained by causality. Next, we guess and construct *causal theoretical models* (CTMs), from which we deduce “*fundamental*” *laws*, define new observables and predict outcomes of new experiments and observations. On the basis of these observations and new experimental outcomes, we improve our initial OMs, modify or replace our old CTMs, make new experiments and gather new observations [12]. During this epistemological cycle, we construct new measuring instruments, the precision of our observation increases and we explore new layers of physical reality (See Figure 38). 

We should not forget that our OMs and CTMs are only mental constructions, providing symbolic mathematical descriptions of natural phenomena. Epistemological questions refer to the knowledge of information gathering used by human beings. From the Bild perspective, it is totally meaningless to even refer to the structure and behavior of a system as such [12].

## 11. Conclusions

Physical reality is a subtle notion. All our science is built on the assumption that there exists an external world governed by some laws of nature which we want to discover and harness. In physics, we construct idealized mathematical models in order to explain, in qualitative and quantitative ways, various phenomena which we observe or create in our laboratories.

Pythagoreans playing with their pebbles understood that numbers were an important abstract notion and believed that the laws of nature could be expressed using them. In particular, by experimenting with strings of different length, they discovered that musical harmony is related to simple whole-number ratios 1:2, 2:3, 3:4… Now we also know that simple fractions describe the symmetry and proportions of a human face and body, 1:3, 1:4, 1:6, 1:8, and 1:10.

As we saw in previous sections, there was a long way from Pythagoreans’ pebbles to quantum mechanics and quarks, but the sacred Pythagorean symbol Tetractys, representing the number “10”, can be easily recognized in the baryon decuplets in the Standard Model. In the binary positional system, all numbers are represented using two digits, “0” and “1”. Computational bases in quantum computing are *n*-dimensional unit vectors.

From Galileo to Einstein, scientists and philosophers were searching for the intelligent design of the universe and constructed sophisticated mathematical models. Einstein asked, “How can it be that mathematics a product of human thought independent of experience is so admirably adapted to the objects of reality?”. Probably, it is less surprising as it seems to be. Man has learned to reason by studying what happens in nature; this is why his reasoning yields the results that accord with nature.

In spite of what some contemporary physicists believe, the law of contradiction appears to be inescapable; the objects do not possess contradictory qualities at the same time. The successes of science were achieved by following this and other Aristotelian principles of reasoning. Moreover, man “has more means at his disposal to make his mathematics fit the physical world. If his “theorems/models” do not fit, he is free to change his axioms/assumptions” [3].

In Mathematics and the Physical World [3], Morris Kline concluded: “Mathematics provides the supreme plan for the understanding and mastery of nature. Mathematics may be the queen of the sciences and therefore entitles to royal prerogatives, but the queen who loses touch with her subjects may lose support and even be deprived of her realm. Mathematicians may like to rise to the clouds of abstract thought, but they should, and indeed they must, return to earth for nourishing food or else die from mental starvation. They are on safer and saner grounds, if they stay close to nature”.

Similar advice can be given to some physicists and philosophers who claim that quantum mechanics proves that an electron can be here and a meter away at the same time, that two perfectly random events in distant locations can be perfectly correlated, that there are millions of parallel worlds or that nature operates according to retro-causality.

Our perceptions are limited and biased by our senses, instruments we construct and by our brains bending our perception of reality to meet our priors, desires or expectations. The stories our brain tells us are influenced by our whole life experiences. It is surprising that we succeeded not only in describing and predicting various phenomena but also created new materials, liberated nuclear energy, landed on the Moon and built ‘quantum computers’.

To explain the invisible world of atoms and elementary particles, we succeeded in creating quantum mechanics, quantum electrodynamics and quantum field theory (QFT), which allowed us to provide a quantitative description of many physical phenomena. Quantum theories are complicated mathematical models, which do not contain intuitive images and explanations as to why observed phenomena and individual experimental outcomes, registered by macroscopic instruments, are produced.

Encouraged by these successes, several scientists believe that when we reconcile general relativity with quantum theory, then we will have the correct quantum theory of everything. In my opinion, we should be much humbler. There is no quantum wave function of the universe and the theory of everything does not exist. Our abstract mathematical models describe only and in an approximate way different layers of physical reality.

Mathematics is a rigorous theory, but often, exact solutions of mathematical equations cannot be found. We encountered this problem when we tried to solve Newton’s equations of motion, Schrodinger equations, interacting quantum field equations, etc. Several macroscopic phenomena can only be studied using chaos theory and catastrophe theory.

QFT requires renormalization and is unable to exactly describe the scattering of bound states. Therefore, semi-empirical models containing several adjustable parameters are added to a theory in order to explain various phenomena in particle physics. In particular, the comparison of the Standard Model with experimental data is a difficult task requiring many free parameters, various phenomenological inputs and a Monte Carlo simulation of events [77,98,99]. The Standard Model also faces serious challenges related to the discovery of black matter, massive neutrinos, tetra-quarks and penta-quarks.

We should not forget that, as Helmholtz, Hertz, Boltzmann and Schrodinger correctly insisted, our models of physical reality are only intellectual constructs of our brain unable to describe nature as it is. For Boltzmann, scientific theories were “metal pictures” having at best a partial similarity to reality. Bohr understood this perfectly and in responding to a question of his colleague said, “There is no quantum world. There is only an abstract quantum mechanical description. It is wrong to think that the task to physics is to find out how the nature is. Physics concerns what we can say about nature”. He also insisted that, “All knowledge presents itself within a conceptual framework adapted to previous experience and any such frame may prove too narrow to comprehend new experience”. Nevertheless, in the phenomena which we observe and create, there should be something behind the scenes which is responsible for their occurrence. In our opinion, quantum probabilities neither correspond to the irreducible propensities of individual physical systems nor to the beliefs of some human agents, but they are the objective properties of quantum phenomena and experiments as a whole.

Bohr often claimed that a more detailed description of quantum phenomena is unnecessary and even impossible. Contrary to Bohr, Einstein believed that there should be some more detailed explanation of quantum probabilities. In spite of what is often believed, the Bohr–Einstein quantum debate cannot be closed [74,75,76]. The loophole-free Bell Tests give additional arguments in favor of Bohr’s contextuality/complementarity, but they proved neither the completeness of quantum mechanics nor its nonlocality [78,79,100,101,102,103,104,105,106,107,108,109,110,111,112]. In fact, we do not even know whether quantum mechanics is predictably complete for the phenomena it wants to describe [74,76,77,100,101,102,103,104,105,106,107,108,109,110,111,112,113,114,115,116,117].

In Bell Tests, we can only assess the plausibility of particular probabilistic models/couplings, and it is true that we may reject the so-called local hidden variable model based on the Bell locality assumption (the assumption which should rather be called non-contextuality) [78,107,108]. This does not the mean that long-range correlations in Bell Tests are due to bizarre influences. Bell Tests cannot reject contextual probabilistic models in which individual binary outcomes in distant laboratories are produced locally in a deterministic way. Moreover, contrary to what many believe, closing the *freedom of choice loophole* in Bell Tests does not close the theoretical *contextuality loophole* [78,102,103]. A true resource for quantum information is entanglement and *contextuality* [118,119].

Only if an experiment is outputting in each trial a triplet or a quadruplet Bell and CHSH inequalities hold for any finite sample. Therefore, if one is analyzing experimental spreadsheets and avoids any metaphysical conclusions, then the violation of Bell and CHSH inequalities by the data, gathered in physics and in social sciences, proves only that the corresponding two column data spreadsheets cannot be reshuffled to form triplets or quadruplets [116,117].

In spite of the fact that QM and QFT are abstract mathematical models, we should not abandon analyzing the metaphysical implications of them. An interesting recent discussion of these implications may be found in [120,121].

As we explained in this article, our successes in harnessing the forces of nature were due to the assumption that behind our imperfect sensorial observations, there is an intelligent design to be discovered. Assuming that there is nothing behind the scenes, and evoking magic to explain some quantum phenomena is not only unjustified but counterproductive.

## Figures and Tables

**Figure A1:**
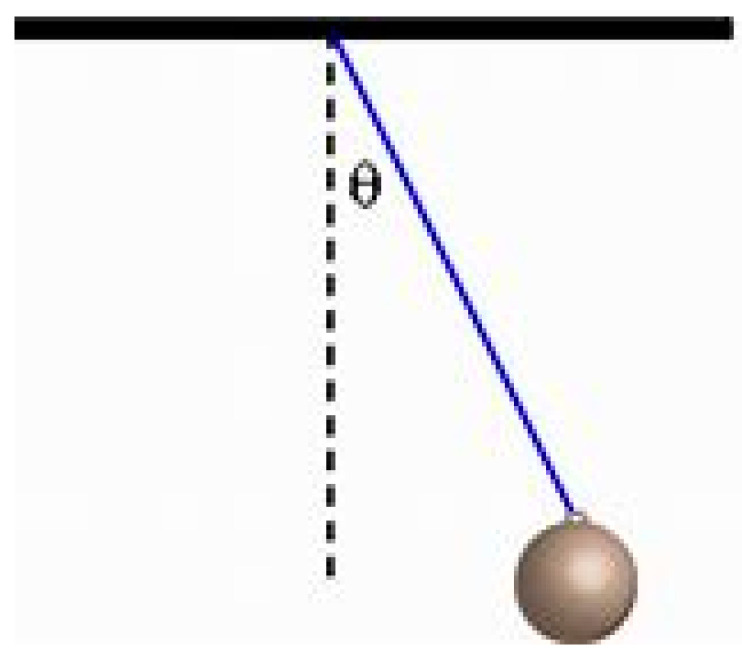
A simple pendulum with one degree of freedom and one generalized coordinate θ.

**Figure A2:**
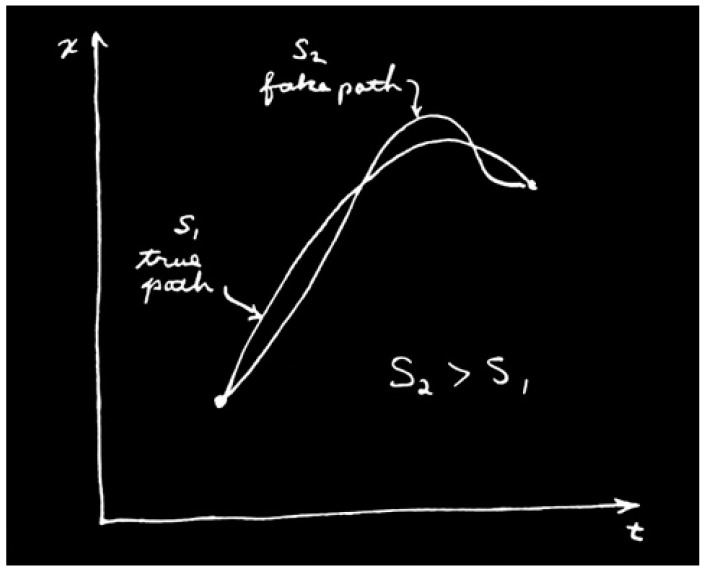
Action S is greater on path 2, in comparison with the path chosen by a material point in the gravitational field on the Earth.

## Appendix A. Lagrangian and Hamiltonian Mechanics

The motion of a planet around the Sun is obtained by solving Newton’s equation for two material points of masses m and M:

(A1) $$m{\ddot{r}}_{1}=G\frac{mM}{|r_{2}-r_{1}{|}^{3}}(r_{2}-r_{1});\;M{\ddot{r}}_{2}=-\;\;G\frac{mM}{|r_{2}-r_{1}{|}^{3}}(r_{2}-r_{1})$$

where G is the universal gravitational constant and *r*_i_ denote 3-dimensional vectors. From (A1) by adding two equations, we obtain that $m{\ddot{r}}_{1}$ + $M{\ddot{r}}_{2}$ = **0** and we find that the total linear momentum ***P*** = m***v*_1_**+ M***v***_2_ is conserved. Next, we define the center of mass position vector ***R*** = (m***r***_1_ + M***r***_2_)**/**(m + M). The center of mass is moving with the constant velocity ***P*/**(m + M). The position vectors of the planet and Sun can be determined using ***R*** and a relative position of the planet with respect to Sun ***r*** = ***r***_1_ − **r**_2_. By subtracting the second equation from the first in (A1), we obtain a simple equation allowing us to determine ***r*** as follows:

(A2) $$\ddot{r}=-G\frac{M+m}{|r{|}^{3}}r$$

Equations (A1) and (A2) completely determine the motion of the planet. Using them, one can also easily demonstrate the conservation of the total energy E, as follows:

(A3) $$E=K+U=\frac{m{{\dot{r}}_{1}}^{2}+M{{\dot{r}}_{2}}^{2}}{2}-G\frac{Mm}{\left|r_{1}-r_{2}\right|}$$

Here, K is the kinetic energy and U is the potential energy. If we choose the origin of the coordinate frame in the center of the mass frame, then Equation (A3) can be rewritten as follows:

(A4) $$E=\frac{\mu {\dot{r}}^{2}}{2}-G\frac{Mm}{\left|r\right|}$$

where a reduced mas $\mu =\frac{mM}{m+M}$.

Another important law is the conservation of the total angular momentum L:

***L*** = ***p***_1_ × ***r***_1_ + ***p***_2_ × ***r***_2_ (A5)

where ***p***_i_ = m_i_ ***v***_i_ are the corresponding individual linear momenta and the “x” denotes the vector product.

It is easy to show that the total energy and the total linear and angular momentum are conserved for any isolated system of N mass points *m_n_*, *n* = 1, … N, evolving under the influence of conservative forces $F_{n}=-\frac{\partial U}{\partial r_{n}}$, where the total potential energy U = U(***r***_1…_***r***_N_).

A motion of a system of N material points can be represented as a motion of one point in a configuration space R^3N^: (***r***_1_, ***r***_2_, …***r***_N_) = x = (x_1_, … x_3N_). If we do not impose any constraints on the motion of N material points, the system has 3N degrees of freedom. In most practical cases, there are constraints imposed. An object has one degree of freedom if it can only slide inside a curved tube in the gravitational field or 2 degrees of freedom if it can slide on the inclined plane. Similarly, a simple pendulum (a suspended small mass m) has one degree of freedom, and its motion is completely determined by one generalized coordinate, an angle θ (See Figure A1).

In general, if we impose several constraints, a system has *s* degrees of freedom and its time evolution can be completely described by generalized coordinates *q* = (*q*_1_, … *q*_s_), describing a hypersurface in the configuration space. If forces depend on time, then this hypersurface is moving inside the configuration space. Thus, after expressing the position vectors and their derivatives in terms of *q* and $\dot{q}$, the kinetic energy $K=K(q,\dot{q})$ and the potential energy U=U(q) can be derived. To find *q*(t) for given initial conditions $q(t_{0})$ and $\dot{q}(t_{0})$, one has to solve the Euler–Lagrange equations [27,28,29]:

(A6) $$\frac{d}{dt}\frac{\partial L}{\partial {\dot{q}}_{i}}-\frac{\partial L}{\partial q_{i}}=0$$

where,

(A7) $$L=K-U=L(q_{1},\ldots q_{s},{\dot{q}}_{1},\ldots {\dot{q}}_{s},t)$$

For one dimensional harmonic oscillator $L=\frac{m{\dot{x}}^{2}}{2}-\frac{kx^{2}}{2}$ and

(A8) $$\frac{d}{dt}\frac{2m\dot{x}}{2}-\frac{-2kx}{2}=m\ddot{x}+kx=0$$

which is Newton’s equation.

Since antiquity, man wanted to maximize the area bounded by a curve within a given perimeter or maximize the volume bounded by a surface within a given area. These and similar problems can be solved using the calculus of variations developed by John Bernoulli, Euler and Lagrange [3,29]. In the 17th century, Pierre de Fermat demonstrated the principle of least time, according to which the light traveling between the two points P and Q takes the path requiring the shortest (extremal) time. It suggested that perhaps this principle can be generalized to include other natural phenomena. In 1744, Pierre de Maupertuis announced that nature always behaves so as to minimize a certain integral called *action.* From this principle, he deduced Newton’s equations of motion and the optical phenomena. He thought that his principle was the scientific proof of the existence of God, for it was, “*so wise a principle as to be worth only of the Supreme Being*” [3]. The principle of the least action was rephrased and generalized by Lagrange, Jacoby and Hamilton [28,29,30]. It can be summarized as follows:

If a system evolves from a point q_1_ = q(t_1_) to another point q_2_ = q(t_2_), under the influence of conservative forces, following the path parametrized by q(t), which is the solution of Equation (A6), then a certain integral S called action remains stationary (δS = 0) for small arbitrary independent changes in the path from q(t) to q(t) + δq(t), such that δq(t_1_) = δq(t_2_) = 0. Action S is usually defined as follows:

(A9) $$S[q,\dot{q},t]=\int_{t_{1}}^{t_{2}}L(q_{1},\ldots q_{s},{\dot{q}}_{1},\ldots {\dot{q}}_{s},t)dt$$

and the variation δS as difference in S up to the first orders of δq and $\delta \dot{q}$:

(A10) $$\delta S=S[q+\delta q,\dot{q}+\delta \dot{q},t]-S[q,\dot{q},t]\approx \int_{t_{1}}^{t_{2}}(\frac{\partial }{\partial q}L\delta q+\frac{\partial }{\partial \dot{q}}L\delta \dot{q})dt=0$$

The action remains stationary for the motion in the configuration space between any two points q_1_ and q_2_ and is the least for close points (short “path”). It can also be proven that, by adding to the original Lagrangian the total derivative of an arbitrary function f(q, t,), one obtains the same solution for the stationary path [28,29].

The mathematical condition δS = 0 chooses from the infinity of possible “evolutions” of the system the evolution consistent with Newton’s equations. Since L = K − U, one can correctly conclude that physical systems, in the field of conservative forces, follow the paths in the configuration space in such a way that the average of the difference between the kinetic energy and potential energy on each segment of the path remains minimal (extremal). (See Figure A2).

One should not forget that the “equivalence” between (A6) and (A10) is the equivalence of two mathematical descriptions and it does not justify teleological speculations.

Nevertheless, the least action principle allows an easy derivation of the Hamilton–Jacoby equations and Hamilton’s equations of motion, which are first order partial differential equations in the newly modified coordinates (q, p) = (q_1_, … q_s_, p_1_, … p_s_), where a generalized momentum $\;p_{i}=\frac{\partial L}{\partial {\dot{q}}_{i}}\;$.

By the introduction of generalized momenta, all information about system evolution is contained in a curve (q(t), p(t)) in a 2s dimensional phase space F.

The important function Hamiltonian is defined as [27,30]:

(A11) $$H(q,p,t)=\sum_{i=1}^{s}p_{i}{\dot{q}}_{i}-L(q_{1},\ldots q_{s},{\dot{q}}_{1},\ldots {\dot{q}}_{s},t)$$

where $\;{\dot{q}}_{i}={\dot{q}}_{i}\;(q,p)$. Using (A6), (A11) and the definition of the generalized momenta we immediately obtain Hamilton equations of motion:

(A12) $${\dot{q}}_{i}=\frac{\partial H}{\partial p_{i}}\;\dot{p}=-\frac{\partial H}{\partial q_{i}}$$

For one dimensional oscillator (A8) we obtain $\frac{\partial L}{\partial \dot{x}}=m\dot{x}=p$ and:

(A13) $$H=p\dot{x}-\frac{m{\dot{x}}^{2}}{2}+\frac{kx^{2}}{2}=\frac{p^{2}}{m}-\frac{p^{2}}{2m}+\frac{kx^{2}}{2}=\frac{p^{2}}{2m}+\frac{kx^{2}}{2}=E$$

where $\frac{p^{2}}{2m}+\frac{kx^{2}}{2}=E$ is a constant energy of the system (because its Lagrangian does not depend on time). Hamilton’s equations of motion are again equivalent to Newton’s equation:

(A14) $$\dot{x}=\frac{\partial H}{\partial p}=\frac{p}{m}\;\dot{p}=-\frac{\partial H}{\partial x}=-kx\;\to \;m\ddot{x}=-kx$$

The trajectory in the space of the system is, in general, an ellipse (see the energy conservation equation) and one can see the animation of this motion, for example, on https://en.wikipedia.org/wiki/Phase_space (accessed on 5 September 2024).

The Hamiltonian equations play an important role in different domains of science including chaos theory, quantum mechanics, quantum field theory and the Standard Model. The canonical quantization consists of the replacement of coordinates and momenta by operators, and the replacement of the Poisson brackets by the commutators (https://en.wikipedia.org/wiki/Poisson_bracket) (accessed on 5 September 2024).
